# Population Pharmacokinetics of Caspofungin and Dose Simulations in Heart Transplant Recipients

**DOI:** 10.1128/aac.02249-21

**Published:** 2022-04-07

**Authors:** Zheng Wu, Jinhua Lan, Xipei Wang, Yijin Wu, Fen Yao, Yifan Wang, Bo-xin Zhao, Yirong Wang, Jingchun Chen, Chunbo Chen

**Affiliations:** a School of Biology and Biological Engineering, South China University of Technology, Guangzhou, Guangdong, China; b Department of Critical Care Medicine, Guangdong Provincial People’s Hospital, Guangdong Academy of Medical Sciences, Guangzhou, Guangdong, China; c Research Center of Medical Sciences, Guangdong Provincial People's Hospital, Guangdong Academy of Medical Sciences, Guangzhou, Guangdong, China; d Guangdong Provincial Key Laboratory of Clinical Pharmacology, Guangdong Cardiovascular Institute, Guangdong Provincial People's Hospital, Guangdong Academy of Medical Sciences, Guangzhou, Guangdong, China; e Department of Intensive Care Unit of Cardiac Surgery, Guangdong Provincial People's Hospital, Guangdong Academy of Medical Sciences, Guangdong Cardiovascular Institute, Guangzhou, Guangdong, China; f Department of Pharmacy, Nanfang Hospital, Southern Medical University, Guangzhou, Guangdong, China; g Department of Intensive Care Unit of Cardiovascular Surgery, Guangdong Cardiovascular Institute, Guangdong Provincial People’s Hospital, Guangdong Academy of Medical Sciences, Laboratory of South China Structural Heart Disease, Guangzhou, Guangdong, China; h Department of Critical Care Medicine, Maoming People’s Hospital, Maoming, Guangdong, China; i The Second School of Clinical Medicine, Southern Medical University, Guangzhou, Guangdong, China

**Keywords:** heart transplantation, caspofungin, pharmacokinetic

## Abstract

The effect of heart transplantation (HTx) on the pharmacokinetics (PK) of caspofungin is not well-characterized. The aim of this study was to investigate the population PK of caspofungin in HTx and non-HTx patients and to identify covariates that may affect the PK of caspofungin. Seven successive blood samples were collected before administration and at 1, 2, 6, 10, 16, and 24 h after the administration of caspofungin for at least 3 days. This study recruited 27 HTx recipients and 31 non-HTx patients with 414 plasma concentrations in total. A nonlinear mixed-effects model was used to describe the population PK of caspofungin. The PK of caspofungin was best described by a two-compartment model. The clearance (CL) and volume of the central compartment (*V_c_*) of caspofungin were 0.385 liter/h and 4.27 liters, respectively. The intercompartmental clearance (*Q*) and the volume of the peripheral compartment (*V_p_*) were 2.85 liters/h and 6.01 liters, respectively. In the final model, we found that albumin (ALB) affected the CL of caspofungin with an adjustment factor of −1.01, and no other covariates were identified. In this study, HTx was not found to affect the PK of caspofungin. Based on the simulations, the dose of caspofungin should be proportionately increased in patients with decreased ALB levels.

## INTRODUCTION

Heart transplantation (HTx) is an effective method to improve the survival of patients with terminal heart failure (1). Invasive fungal infection is an infrequent but serious complication after HTx (2–5). Antifungal prophylaxis in HTx recipients may reduce the incidence of fungal infections and the associated mortality (6). In a previous study, caspofungin was found to be an effective antifungal agent in heart and lung transplant recipients with invasive aspergillosis (7). Studies have also demonstrated the efficacy of caspofungin in the treatment of invasive candidiasis (8, 9). Moreover, caspofungin is currently suggested as the first-line therapy for invasive candidiasis (10, 11). Caspofungin specifically inhibits the synthesis of β-1,3-d-glucan in the cell wall of fungi but does not injure mammalian cells (12).

After HTx, patients need to be monitored in the surgical intensive care unit (SICU). Renal complications (13) and low-output syndrome (14) are common following HTx and may cause the change of the pharmacokinetics (PK) of caspofungin. We sought to assess whether there is a difference in the PK of caspofungin between HTx patients and non-HTx patients. Renal impairment is a frequent occurrence in critically ill patients (15), and it is not easy to predict acute kidney injury (16–19). In addition, various other factors affect drug absorption, distribution, and metabolism (20–23). Standard dosing of caspofungin in critically ill patients was associated with low drug exposure and suboptimal therapeutic effects (24). Studies have identified several variables that may affect the PK parameters of caspofungin in critically ill patients, such as body weight and serum albumin (ALB) concentration (25–28). To the best of our knowledge, no study has reported the PK of caspofungin in HTx recipients. This prospective, single-center, open-label research aimed to investigate the PK of caspofungin in HTx recipients and to identify the factors that affect PK parameters.

## RESULTS

### Patient characteristics.

A total of 58 patients and 414 plasma concentrations were included in the study. We divided these patients into two groups. A total of 27 patients (22 males and 5 females; median age, 50 years) who received HTx were recruited in this study as the HTx group, and 31 (21 males and 10 females; median age: 58 years) critically ill patients who had not received HTx were enrolled as the control group. The demographic and clinical characteristics of the study population are summarized in Table 1. The median (range) extracorporeal membrane oxygenation (ECMO) blood flow rate was 2.8 liters/min (2.2 to 3.5 liters/min). The median (range) continuous renal replacement therapy (CRRT) blood flow rate was 200 mL/min (200 to 220 mL/min).

**TABLE 1:** Demographic characteristics of the study population^*a*^

Parameter	Heart transplantation group (*n* = 27)	Control group (*n* = 31)
Age (yr)	50 (20 to 73）	58 (22 to 78)
Male/female, n (%)	22/5 (81/19)	21/10 (68/32)
Weight (kg)	59.5 (43.5 to 76)	62.0 (48.0 to 100.0)
Height (cm)	168 (156 to 178)	165 (141 to 178)
Body mass index (kg/m^2^)	20.8 (17.0 to 27.8)	23.4 (15.8 to 34.6)
Alanine aminotransferase (U/liter)	17 (5 to 3,587)	23 (1.8 to 3,201)
Aspartate aminotransferase (U/liter)	38 (9 to 5,007)	60 (14 to 2,463)
Total bilirubin (μmol/liter)	19.5 (8.5 to 183.1)	32.4 (11.6 to 375.6)
Direct bilirubin (μmol/liter)	8.0 (2.7 to 107.9)	13.7 (1.5 to 311.3)
Total protein (g/liter)	64.7 (49.4 to 89.6)	61.1 (48.6 to 91.4)
Albumin (g/liter)	40.3 (29.7 to 49.3)	33.7 (26.41 to 48.40)
Platelet count (×10^9^/liter)	74 (2 to 222)	104 (25 to 337)
Procalcitonin (μg/liter)	5.58 (0.05 to 149.49)	3.99 (0.64 to 118.93)
Serum creatine (μmol/liter)	204.9 (13.6 to 626.2)	184.12 (34.45 to 620)
Creatinine clearance (mL/min)	23.3 (5.7 to 169.0)	24.06 (6.38 to 149.6)
CRRT (yes/no), n (%)	12/15 (44/56)	16/15 (52/48)
CRRT blood flow rates (mL/min)	200 (200 to 210)	200 (200 to 220)
ECMO (yes/no), n (%)	8/19 (30/70)	8/23 (26/74)
ECMO blood flow rates (liters/min)	2.75 (2.3 to 3.5)	2.85 (2.2 to 3.2)
SOFA (score)	9 (1/16)	10 (5/16)

*^a^*The data are presented as median (range) or frequency (percentage), unless indicated otherwise. CRRT, continuous renal replacement therapy; ECMO, extracorporeal membrane oxygenation; SOFA, sequential organ failure assessment.

### Establishment and evaluation of a pharmacokinetic model.

After analysis of one-, two, and three-compartment models, the time course of plasma caspofungin concentrations was found to be best described by a two-compartment model with an additive residual error of 0.213 mg/liter and a proportional error of 13.4%. The population PK parameter estimates with 95% confidence intervals (CI) based on bootstraps are listed in Table 2. ALB was identified as a significant covariate for clearance (CL), leading to a drop in objective function value (OFV) of −14.674. No other significant covariates were found for PK parameters in our study, and the relationship between the covariates and PK parameters can been found in Fig. S2.

**TABLE 2:** The estimates of the parameters in the caspofungin final pharmacokinetic model^*a*^

Parameters	Estimates	RSE (%)	Median by 1,000 bootstraps	95% CI by 1,000 bootstraps
CL (liter/h)	0.385	5	0.383	0.349 to 0.422
*V_c_* (liter)	4.27	12	4.28	2.97 to 5.20
*Q* (liters/h)	2.85	11	2.79	2.13 to 4.57
*V_p_* (liter)	6.01	13	6.02	5.08 to 7.34
Θ_ALB-CL_	−1.01	14	−1.09	−1.90 to 0.18
CL_INTER VAR_ (%)	33.5	13	32.7	25.6 to 43.0
*V_c_*_INTER VAR_ (%)	67.5	14	66.6	52.1 to 85.5
*V_p_*_INTER VAR_ (%)	47.7	17	46.5	9.8 to 75.7
*Q*_INTER VAR_ (liter/h)	0 FIX			
Additive error (mg/liter)	0.213	29	0.211	0.069 to 0.391
Proportion error (%)	13.4	11	12.9	8.2 to 16.9

*^a^*CI, confidence interval; CL_INTER VAR_, the interindividual variability of CL; Q, intercompartmental clearance; *Q*_INTER VAR_, interindividual variability of *Q*; RSE, relative standard error; *V_c_*, volume of distribution of the central compartment; *V_c_*_INTER VAR_, the interindividual variability of *V_c_*; *V_p_*, volume of distribution of the peripheral compartment; *V_p_*_INTER VAR_, interindividual variability of *V_p7_.*

**FIG 1:**
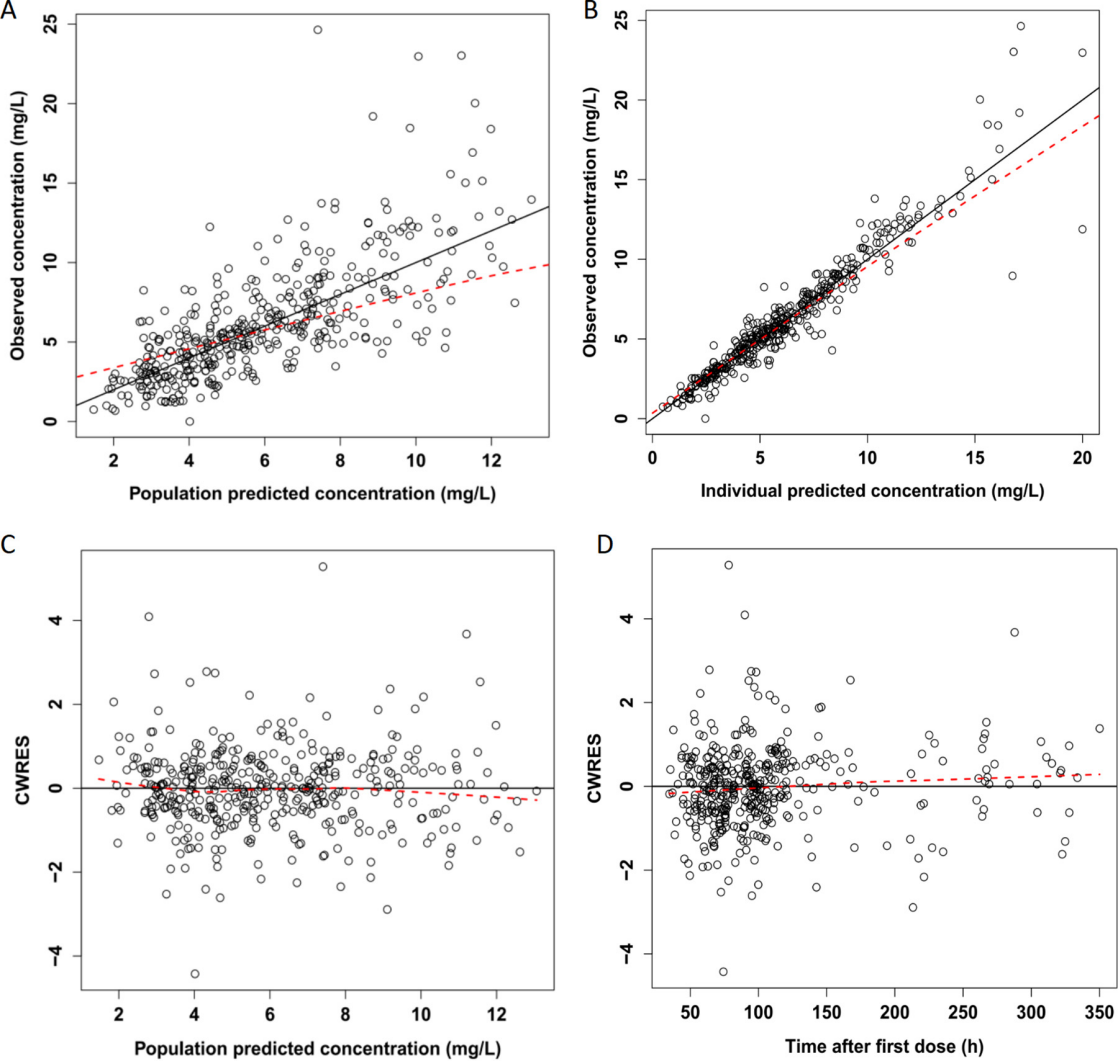
Goodness-of-fit (GOF) plots of the final model. (A) Observed concentration versus population predicted concentration. (B) Observed concentration versus individual predicted concentration. (C) Conditional weighted residuals (CWRES) versus population predicted concentration. (D) CWRES versus time after first dose.

**FIG 2:**
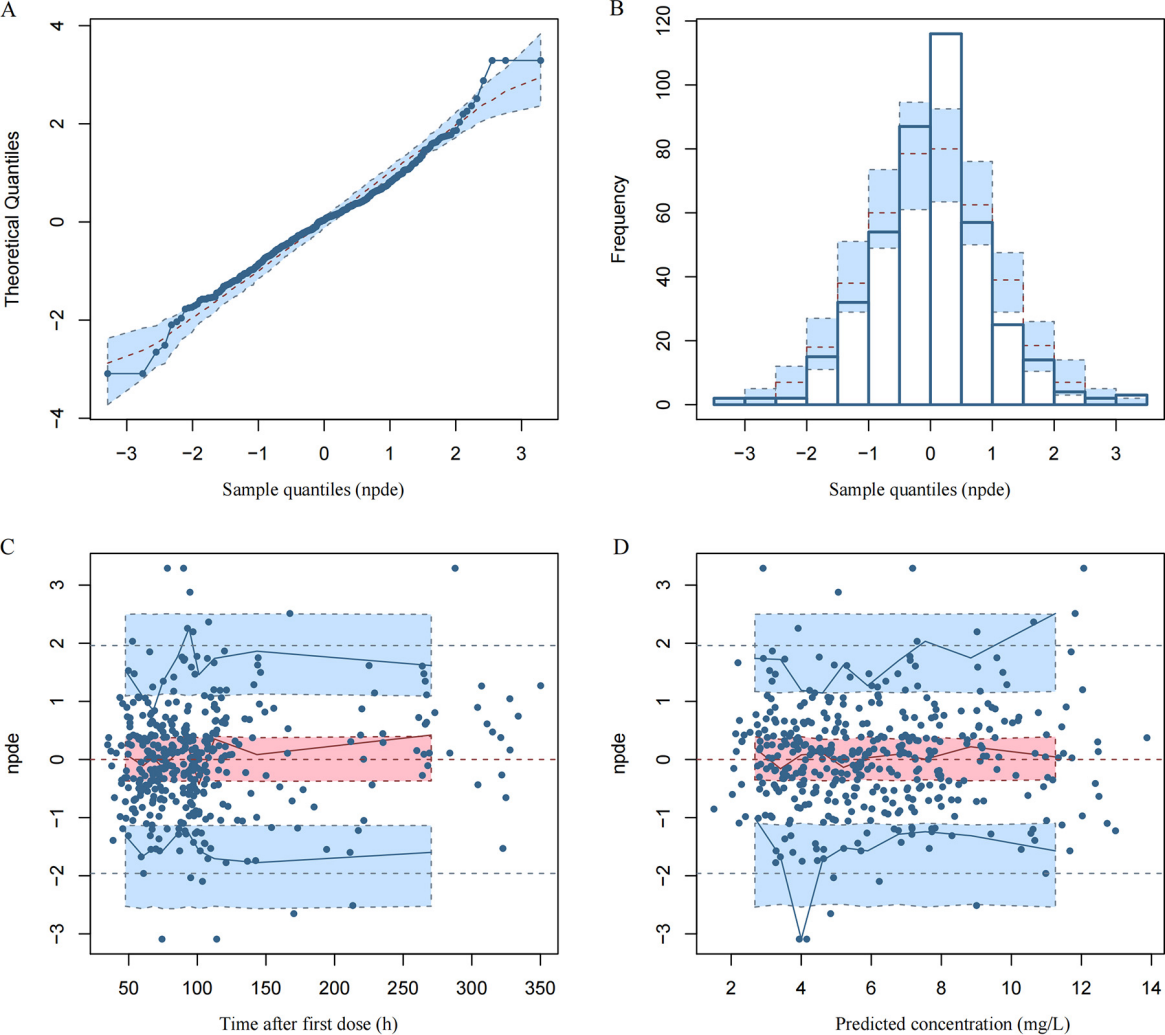
Normalized prediction distribution error (NPDE) metrics for the final PK model. (A) Quantile-quantile plot of NPDE. (B) Distribution of NPDE. (C) NPDE versus time. (D) NPDE versus predicted concentrations. The observed concentrations are shown as filled circles, and solid lines represent the 5th, 50th, and 95th percentiles of observed data. Red- or blue-shaded areas represent the 95% prediction interval.

**FIG 3:**
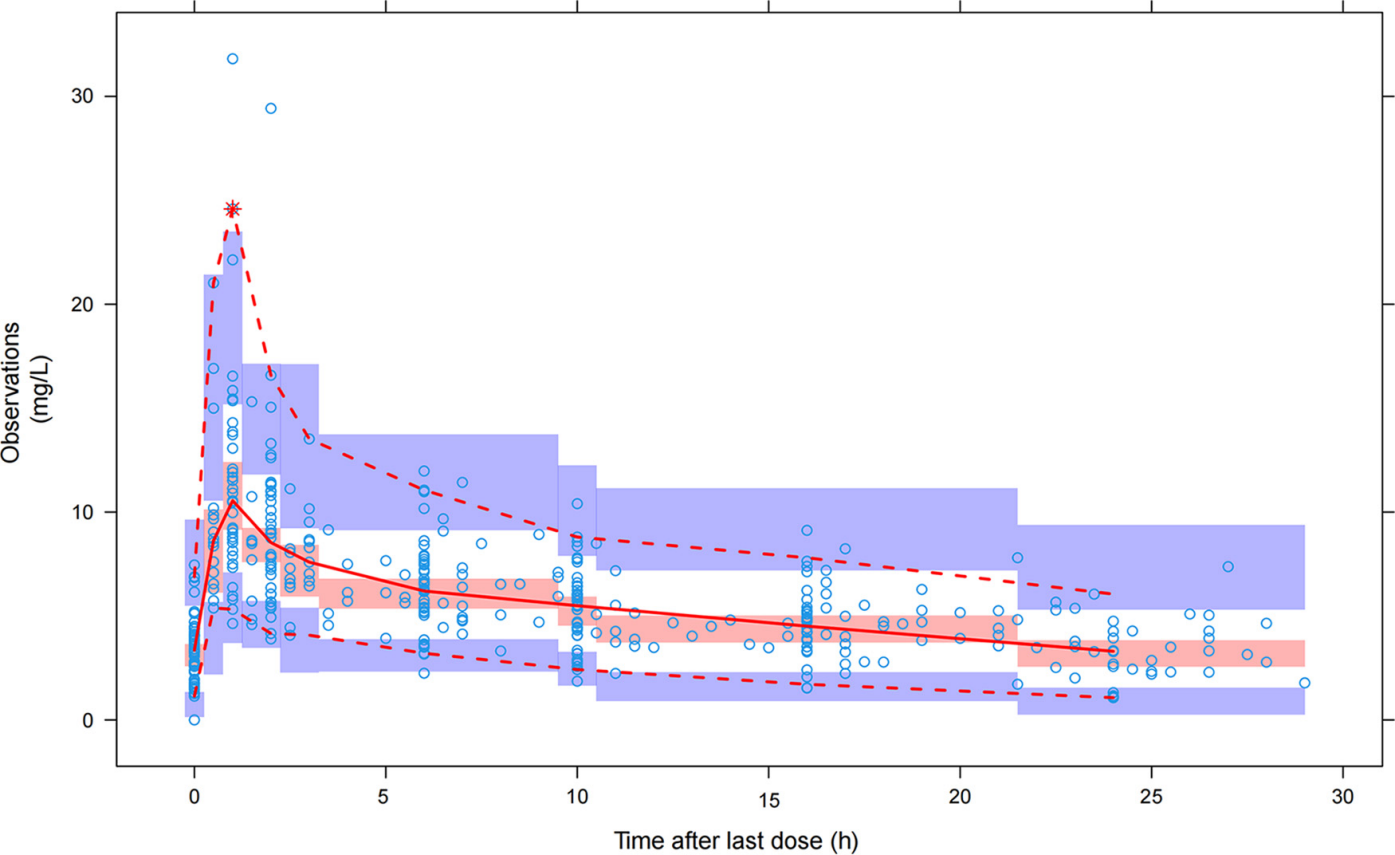
Visual predictive check (VPC) for the final pharmacokinetic model of caspofungin. The open circles represent the observed caspofungin concentrations. The middle solid, lower dashed, and upper dashed lines represent the median, 5th, and 95th percentiles for the observed data, respectively. The shaded areas represent the 95% CI for the simulated predicted median and 5th and 95th percentiles constructed from simulated data sets of individuals from the original data set.

Goodness-of-fit (GOF) plots are displayed in Fig. 1. In 1,000 simulations, the distribution of normalized prediction distribution error (NPDE), with a mean of 0.01159 and a variance of 0.8501, was normal, which indicated good predictive value of the final model (Fig. 2). The final model was confirmed by visual predictive check (VPC) plots and 1,000 iterations of bootstrap. As shown in the VPC plots (Fig. 3), most of the observed concentration data were included in the 95% prediction interval of the simulated data, and the parameters of the final model were similar to the medians generated by the bootstraps. All these results indicated the high accuracy and stability of the final model.

### Pharmacokinetic parameters of caspofungin.

The median (range) CL in the HTx group and control group were 0.35 liter/h (0.18 to 0.98 liter/h) and 0.45 liter/h (0.22 to 0.84 liter/h), respectively. The median (range) central compartment distribution volume (*V_c_*), interventricular space (*Q*), and peripheral compartment distribution volume (*V_p_*) of the HTx group were 3.14 liters (1.35 to 10.02 liters), 2.85 liters/h, and 4.98 liters (1.52 to 8.59 liters ), respectively. The *V_c_*, *Q*, and *V_p_* of the control group were 5.27 liters (0.81 to 14.03 liters), 2.85 liters/h, and 6.31 liters (4.07 to 11.73 liters), respectively. The median (range) area under the curve (AUC) values in the HTx group and control group were 126.86 mg·h/liter (26.78 to 292.27 mg·h/liter) and 105.64 mg·h/liter (52.21 to 217.99 mg·h/liter), respectively. The results indicated a significant difference between the two groups with respect to CL and AUC (*P < *0.05) (Table 3).

**TABLE 3:** Pharmacokinetic characteristics of caspofungin in HTx and non-HTx patients^*a*^

Parameter	Heart transplantation group (*n* = 27)	Control group (*n* = 31)	*P*
*C*_max_, mg/liter	11.74 (5.39 to 24.64)	7.89 (5.01 to 16.92)	0.004
*C*_min_, mg/liter	3.59 (0.75 to 6.34)	2.92 (0.80 to 5.45)	0.091
*V_c_*, liter	3.14 (1.35 to 10.02)	5.27 (0.81 to 14.03)	0.001
*V_p_*, liter	4.98 (1.52 to 8.59)	6.31 (4.07 to 11.73)	0.001
CL, liter/h	0.35 (0.18 to 0.98)	0.45 (0.22 to 0.84)	0.046
AUC, mg·h/liter	126.86 (26.78 to 292.27)	105.64 (52.21 to 217.99)	0.042

*^a^*The data are presented as medians (range). AUC, area under the concentration-time curve; CL, clearance; *C*_max_, peak plasma concentration; *C*_min_, through plasma concentration; HTx, heart transplantation; *V_c_*, volume of distribution of the central compartment; *V_p_*, volume of distribution of the peripheral.

### Simulations.

**FIG 4:**
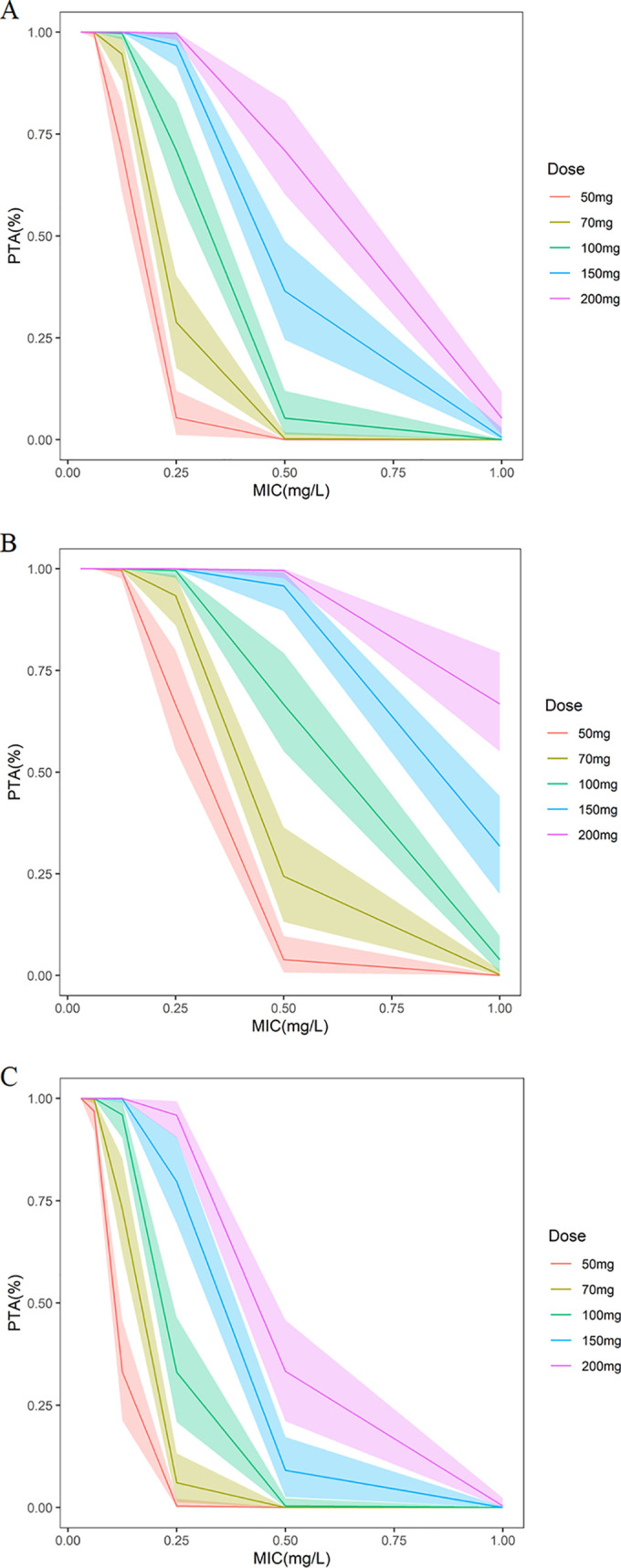
Probability of target attainment (PTA) of caspofungin versus C. albicans (A), C. glabrata (B), and C. parapsilosis (C) at doses of 50 to 200 mg (1-h infusion) once daily in the patients with albumin of 37 g/liter. The shading around the lines represents the 95% confidence intervals of the prediction. MIC, minimum inhibitory concentration.

The probability of target attainment (PTA) values based on different dose regimens and minimum inhibitory concentrations (MICs) with ALB levels of 37 g/liter are shown in Fig. 4. The results showed that a common maintenance dose of 50 mg caspofungin can obtain excellent PTA in patients for Candida spp. when MIC was 0.06 mg/liter or less. However, the common maintenance dose of 50 mg is not suitable for all situations. When patients infected by Candida glabrata and MIC 0.25 mg/liter or less, the recommended maintenance dose is 50–100 mg (Fig. S1). For Candida albicans, the recommended maintenance dose is 50 to 200 mg when MIC is 0.25 mg/liter or less. For patients infected by Candida parapsilosis (MIC of 0.125 mg/liter or less), the recommended maintenance dose is 50 to 150 mg. For patients infected by C. parapsilosis (MIC was 0.25 mg/liter) and ALB of 30 g/liter or more, the recommended maintenance dose is 200 mg. More recommended dose regimens can be found in Fig. 4 and Fig. S1.

## DISCUSSION

To the best of our knowledge, this is the first population PK analysis of caspofungin for HTx recipients. Our results suggest that HTx may not significantly affect the PK of caspofungin. However, we observed a significant difference in CL between the two groups. The possible reason for this difference was ALB levels. The ALB level in the HTx group was 40.3 g/liter (29.7 to 49.3 g/liter), while the ALB level in the control group was 33.7 g/liter (26.41 to 48.40 g/liter). Our finding suggests that ALB may associate with the CL of caspofungin.

Weight was one of the most important covariates that may affect the PK parameters of caspofungin. Several previous studies have identified weight as an important covariate that affects CL and *V* of caspofungin (24, 25, 29). For example, Hall et al. (29) showed that the *V* and CL of caspofungin increased with an increase in weight. Würthwein et al. (30) found that the weight was most suitable as a covariate for caspofungin CL and *V*. Märtson et al. (25) proposed a weight-based dose regimen for caspofungin. However, several other studies (26, 31–33) have found no significant effect of weight on the pharmacokinetic parameters of caspofungin, similar to our research. Our study had a small sample size and a small range of changes in body weight. The median (range) weight was 60 kg (43.5 to 100 kg). Therefore, the impact of weight on PK parameters may have been underestimated because of the small individual differences.

In our study, ALB was found to affect the PK of caspofungin, consistent with earlier findings (26, 33). Kurland et al. (26) found a negative correlation between hypoalbuminemia and elimination rate constant. Li et al. (33) investigated 42 ICU patients in China who received caspofungin and found that ALB was a factor that affected CL. They also identified total bilirubin (TBIL) as another covariate. ALB and TBIL levels were collectively found to affect CL of caspofungin. In our study, TBIL resulted in an OFV drop of −11.93. However, TBIL did not explain the interindividual variability of CL further after incorporation of ALB in the model. This may be attributable to potential collinearity between the two covariates. Finally, we kept ALB in the model as it caused a greater decrease in OFV. Ulldemolins et al. (34) extensively reviewed the effects of hypoalbuminemia on antibacterial dosing in critically ill patients and concluded that the influence of hypoalbuminemia on antibiotic PK in critically ill patients may be clinically important. The effect of hypoalbuminemia on PK is due to the decrease in the degree of binding of antibiotics to ALB, thereby increasing the unbound fraction of the drug. The unbound fraction is the only fraction available for distribution and clearance from the plasma (central compartment). Hence, hypoalbuminemia is likely to increase the CL of a drug, which would lead to lower antibacterial exposure that may influence the attainment of pharmacodynamic targets (34). For highly protein-bound drugs, such as caspofungin (97% protein binding rate under normal plasma protein levels) (35), changes in ALB level can significantly change the CL of caspofungin.

Several recent studies have shown that ECMO is associated with significant PK changes (36, 37). We also examined ECMO as a covariate and found no significant effect of ECMO on the PK of caspofungin. The same conclusion was reached by a previous study (38) by Wang et al., who investigated the PK of caspofungin in lung transplant recipients on ECMO therapy. This is potentially attributable to the fact that the ECMO circuit may not sequester caspofungin *in vivo*. We also investigated CRRT, and the CRRT blood flow rate and dialysate had no effect on the PK of the caspofungin. The results showed that CRRT did not affect the PK of caspofungin. This is consistent with the observations of Roger et al., who reported no need for changing the dose of caspofungin for patients undergoing either form of CRRT (39). Other factors such as AST and ALT level showed no influence on PK of caspofungin.

The present research used Matlab to simulate different MIC and ALB levels and investigated the PTA with different dose regimens. For patients infected by C. albicans (MIC of 0.06 mg/liter or less), C. glabrata (MIC of 0.125 mg/liter or less), and C. parapsilosis (MIC of 0.03 mg/liter or less), the recommended maintenance dose of 50 mg can obtain a high PTA. This is consistent with previous studies (27, 33). For patients infected by C. parapsilosis and MIC of 0.5 mg/liter or more, there was no appropriate dose regimen in our simulations. In that case, whether to increase the dose of caspofungin or alternative medicine requires further research.

Some limitations of our study need to be considered. First, this was a single-center research with a relatively small sample size, which may affect the generalizability of our results. However, despite the lack of a large sample, our model accurately predicted the caspofungin concentrations in HTx recipients and the control group. Second, we did not assess the pharmacodynamic aspects of caspofungin treatment and could not produce meaningful clinical outcome measurements from a small sample. Therefore, multicenter studies with a larger sample of HTx recipients are required to provide more robust evidence. Third, in the population fit (Fig. 1B), it can be observed that higher concentrations were not well-predicted. The bias of the prediction of the higher concentrations (more than approximately 15 mg/liter) might be caused by the small number of concentrations. ALB can reduce between-subject variability (between the model with covariates and without covariates) from 42.1 to 33.5%. The amount of decline is not large, indicating that there are other unknown covariates that were not reflected in the study, and more research is needed in the future.

In conclusion, this study represents the first step to characterize the PK of caspofungin in HTx recipients. Our results showed no significant effect of HTx on caspofungin PK. According to the final model, ALB level was a significant determinant of the PK of caspofungin. The dose of caspofungin should be proportionately increased in patients with decreased ALB levels.

## MATERIALS AND METHODS

### Study design.

This was a prospective, single-center, open-label study. We recruited patients who underwent HTx at the Guangdong Provincial People’s Hospital in the SICU from July 2019 to July 2021. The inclusion criteria were: (i) patients aged 18 years or older who received caspofungin in SICU and accepted cardiac surgery and (ii) acceptability of multiple blood sampling. Patients who were allergic to caspofungin, those receiving drugs that can affect the PK of caspofungin, and those who could not receive multiple blood sampling were excluded. Patients who underwent HTx in SICU were recruited as the HTx group. Patients who never received HTx but underwent cardiac surgery and being in SICU were recruited in the control group. This study was approved by the Ethics Committee of Guangdong Provincial People’s Hospital, Guangdong Academy of Medical Sciences (approval number GDREC2018355(R1)H).

### Demographic characteristics and data collection.

The clinical data, including age, weight, height, sex, alanine aminotransferase (ALT), aspartate aminotransferase (AST), total bilirubin (TBIL), direct bilirubin (DBIL), total protein (TP), albumin (ALB), serum creatinine (sCr), platelet count (PLT), procalcitonin (PCT), and creatinine clearance (CrCL) were evaluated on the day of sample collection. Other potential covariates included CRRT, ECMO, and baseline sequential organ failure assessment (SOFA) scores. The components of the SOFA scores were collected from medical records.

### Blood sample collection.

Caspofungin (Laboratoires Merck Sharp & Dohme Chibret, France) was administered by 1-h intravenous infusion at a dose of 50 mg every 24 h after a loading dose of 70 mg. Blood samples were collected before administration and at 1, 2, 6, 10, 16, and 24 h after the administration of caspofungin for at least 3 days. Plasma was separated by centrifugation of blood samples at 2,000 × *g* for 10 min at 4°C and stored at −80°C before analysis.

### Analytical procedures.

All plasma samples were detected by the liquid chromatography-tandem mass spectrometry (LC-MS/MS) method. First, 200 μL of acetonitrile-dissolved extraction working solution was added to 100 μL of plasma sample and mixed by vortexing at 3,000 rpm for 1 min. After centrifugation at 14,000 rpm for 15 min at 4°C, 2 μL supernatant was injected for LC-MS/MS analysis. The chromatographic column was an Agilent poroshell 120 SB-C18 (2.7 μm, 3.0 × 50 mm) column. The mobile phase was acetonitrile with 0.1% formic acid (A) and water with 0.1% formic acid (B). In the initial 0 to 0.5 min, the mobile phase was composed of 95% B and 5% A, and then within 0.5 to 1.5 min, the mobile phase B decreased linearly from 95% to 5% and remained unchanged for 1.5 to 2 min. Finally, it returned to 95% B and 5% A, and the entire analysis time was 5 min. The flow rate was 0.5 mL/min, and the column temperature was 35°C. A positive ion mode multiple reaction monitoring detection mode through electrospray ionization was performed with the transitions of *m*/*z* 547.3 → 538.2 for caspofungin and *m*/*z* 549.3 → 540.3 for caspofungin-d4 (IS). The linear range of the method was 0.4 to 25 mg/liter. The intraday and interday precision were 1.56 to 3.32% and 3.40 to 7.48%, respectively, and the deviation of accuracy was 0.96 to 2.21%.

### Population PK model.

We analyzed population PK data using the nonlinear mixed-effects modeling software NONMEM 7.3 (version 7.3, ICON plc, NY, USA). We employed one-, two-, and three-compartment pharmacokinetic models to fit the data and selected the most suitable basic structural model. Further, all potential covariates for caspofungin PK parameters reported in previous studies were analyzed. The covariates included demographic characteristics, renal function indices, liver function indices, HTx status, ECMO, and CRRT. Different covariate types have different variable models. Discrete covariates (such as sex) were modeled as follows.

(1) $$C_{\mathrm{ij}}=C_{\mathrm{tv},j}\times \theta_{j}^{\mathrm{cov}}\times e_{}^{\mathrm{nj}}$$

For continuous covariates (such as weight), an exponential model was selected with average covariates values and an adjusting factor.

(2) $$C_{\mathrm{ij}}=C_{\mathrm{tv},j}\times {\left(\frac{\text{cov}}{{\text{cov}}_{\text{ave}}}\right)}^{\theta_{j}}\times e^{\eta_{j}}$$

In the above equations, *C_ij_* is the individual value. *C_tv,j_* is the population typical value. cov in equation 1 is expressed as 0 or 1, cov in equation 2 is the value of the covariate, and θ_*j*_ is an impact factor. cov_ave_ is the average value of a continuous covariate. Interindividual variability is shown as *η_j_*, which is normally distributed with mean 0 and variance ω^2^. Residual variability was modeled using a mixture of additive and proportional error models.

The first-order conditional interaction estimation method with interaction was used for the analysis. The asymptotic distribution of the change in the OFV is χ^2^. For the forward stepwise covariate evaluation, it was considered significant when the OFV was reduced by at least 3.84 (α = 0.05, 1 degree of freedom); for the backward covariate elimination, it was considered significant when the OFV was increased by at least 10.82 (α = 0.001).

In our study, GOF plots, bootstrap, visual predictive check (VPC), and NPDE were used for model validation. VPC and bootstrap were conducted on Pearl-speaks-NONMEM version 4.8.0 (Uppsala University, Sweden). The GOF plots and NPDE statistics were generated using R and the add-on package NPDE version 2.0 (40). Bootstrap resampling was performed randomly 1,000 times in the original data set, and VPC simulated the final model to simulate 1,000 virtual data. These data were used to test the stability and predictive performance of the model. NPDE also utilized simulation technology to conduct 1,000 simulations based on the final model to assess the data error trends and conduct a normal distribution test.

### Simulations.

This study used Matlab software for simulations. The simulated data were based on the virtual patients with different ALB levels (25, 30, 37, 45, and 50 g/liter) at various dosage regimens (50-, 70-, 100-, 150-, and 200-mg maintenance dose). The median ALB level of the research population was 37 g/liter. We generated 1,000 virtual patients whose pharmacokinetic parameters were randomly assigned according to the distributions with the median of CL, θ_ALB-CL_, and CL interindividual variations and their calculated standard deviations from bootstraps (Table 2) for the simulations. AUC was calculated as dose divided by clearance. Each situation will get 1,000 PTA curves. From these PTA curves, we obtained 95% CI from the 2.5 and 97.5% percentiles (41). The AUC/MIC ratio was considered an indicator of the curative effect of caspofungin. The target AUC/MIC values for C. albicans, C. glabrata, and C. parapsilosis were greater than 865, 450, and 1,185, respectively (42). The tested MICs were 0.03, 0.06, 0.125, 0.25, 0.5, and 1 mg/liter. The Matlab code for simulations can been found in the supplemental materials.

### Statistical analysis.

Statistical analysis was performed using SPSS version 23.0 (SPSS Inc., Chicago, IL). Population PK analyses were performed with the software NONMEM (version 7.3, ICON plc, New York, NY, USA). Simulation was performed using Matlab R2017b (version 9.3, MathWorks, Inc., USA). A Fortran compiler was adopted, and the runs were executed on Pirana (version 2.9.0). Categorical data are expressed as frequency (percentages) and continuous data are presented as medians (range). χ^2^ test, Fisher’s exact test, and *t* test were used to evaluate the differences between the HTx group and control group.

### Data availability.

The raw data analyzed during the current study are available from the authors on reasonable request.
