# Supplementary material for: Population Pharmacokinetics of Caspofungin and Dose Simulations in Heart Transplant Recipients
Source: Antimicrob Agents Chemother. 2022 Apr 7;66(5):e02249-21. doi: 10.1128/aac.02249-21 (PMC9116478; doi:10.1128/aac.02249-21)
Supplement: Supplemental file 1 — Code for simulations and Fig. S1 and S2. Download aac.02249-21-s0001.pdf, PDF file, 13.9 MB [file aac.02249-21-s0001.pdf]

Supplementary data

File for NONMEM

```
#------------------------------
$SUBROUTINES ADVAN3 TRANS4
;---------------------------------
$PK
;---------------------------------
CL = THETA (1) * EXP (ETA (1)) *(ALB/37.42) **THETA (7)
V1 = THETA (2) * EXP (ETA (2))
Q = THETA (3) * EXP (ETA (3))
V2 = THETA (4) * EXP (ETA (4))
S1 = V1
;---------------------------------
$THETA
(0, 0.385,10); CL
(0, 4.27,20); V1
(0, 2.85,10); Q
(0, 6.01,20); V2
(0, 0.134); exponential error
(0, 0.213); additive error
(-3, -1.01,0); ALB
$OMEGA
  0.112; IIV/BSV CL
  0.455; IIV/BSV V1
  0 FIX; IIV/BSV Q
  0.22; IIV/BSV V2
$SIGMA
  1 FIX; residual variability
$ERROR
;---------------------------------
IPRED = F
IRES = DV-IPRED
W = IPRED*THETA (5) +THETA (6)
IF (W.EQ.0) W = 1
IWRES = IRES/W
Y= IPRED+W*ERR (1)
;---------------------------------
$EST METHOD=1 INTERACTION MAXEVAL=9999 SIG=3 PRINT=5 NOABORT POSTHOC
$COV PRINT=E UNCONDITIONAL MATRIX=S
```

File for Matlab

```
nSamples = 1000;
Dose1 = 50;
Dose2 = 70;
Dose3 = 100;
Dose4 = 150;
Dose5 = 200;
ALB = 50;
% Based on Table 2 data
mean_teta_CL = 0.383;
mean_omega_CL = sqrt(log((32.7/100)^2+1));
mean_ALB_CL = -1.09;
std_teta_CL = abs((0.349-0.422)/2/1.64);
std_omega_CL = abs((sqrt(log((43/100)^2+1))-sqrt(log((25.6/100)^2+1)))/2/1.64);
std_ALB_CL = abs((-1.9-0.18)/2/1.64);
% Sample over bootsrap uncertainity
teta_CL_i = normrnd(mean_teta_CL,std_teta_CL,nSamples,1);
omega_CL_i = normrnd(mean_omega_CL,std_omega_CL,nSamples,1);
teta_ALB_CL_i = normrnd(mean_ALB_CL,std_ALB_CL,nSamples,1);
MIC = [0.03 0.06 0.125 0.25 0.5 1];
PTA1 = zeros(nSamples,length(MIC));
PTA2 = zeros(nSamples,length(MIC));
PTA3 = zeros(nSamples,length(MIC));
PTA4 = zeros(nSamples,length(MIC));
PTA5 = zeros(nSamples,length(MIC));
for z = 1:nSamples
AUC1=Dose1./(teta_CL_i(z).*(ALB/37.42).^teta_ALB_CL_i(z).*exp(normrnd(0,omega_CL_i(z),
nSamples,length(MIC))));
AUC2=Dose2./(teta_CL_i(z).*(ALB/37.42).^teta_ALB_CL_i(z).*exp(normrnd(0,omega_CL_i(z),
nSamples,length(MIC))));
AUC3=Dose3./(teta_CL_i(z).*(ALB/37.42).^teta_ALB_CL_i(z).*exp(normrnd(0,omega_CL_i(z),
nSamples,length(MIC))));
AUC4=Dose4./(teta_CL_i(z).*(ALB/37.42).^teta_ALB_CL_i(z).*exp(normrnd(0,omega_CL_i(z),
nSamples,length(MIC))));
AUC5=Dose5./(teta_CL_i(z).*(ALB/37.42).^teta_ALB_CL_i(z).*exp(normrnd(0,omega_CL_i(z),
nSamples,length(MIC))));
PTA1(z,:)= mean((AUC1./(ones(nSamples,1)*MIC))>1185);
PTA2(z,:)= mean((AUC2./(ones(nSamples,1)*MIC))>1185);
PTA3(z,:)= mean((AUC3./(ones(nSamples,1)*MIC))>1185);
PTA4(z,:)= mean((AUC4./(ones(nSamples,1)*MIC))>1185);
PTA5(z,:)= mean((AUC5./(ones(nSamples,1)*MIC))>1185);
end
prctPTA1 = prctile(PTA1,[5 50 95]);
prctPTA2 = prctile(PTA2,[5 50 95]);
```

```
prctPTA3 = prctile(PTA3,[5 50 95]);
prctPTA4 = prctile(PTA4,[5 50 95]);
prctPTA5 = prctile(PTA5,[5 50 95]);
```

A

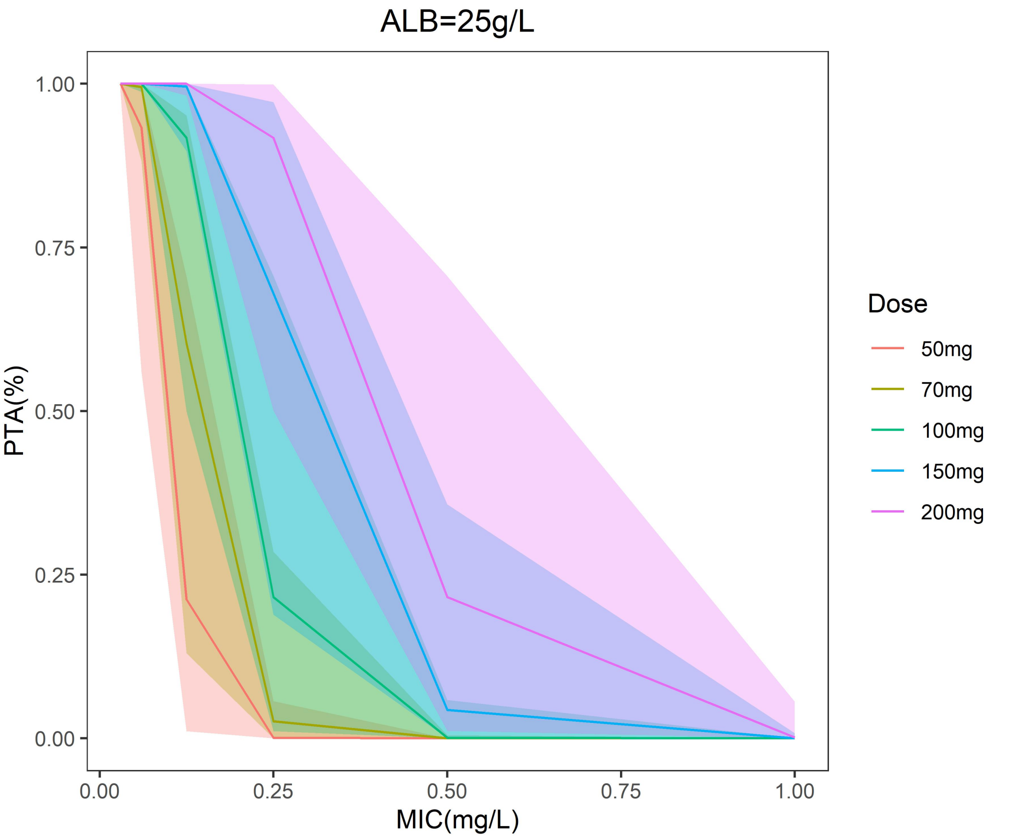
ALB=25g/L
PTA(%)
1.00
0.75
0.50
0.25
0.00
0.00
0.25
0.50
0.75
1.00
MIC(mg/L)
Dose
50mg
70mg
100mg
150mg
200mg

B

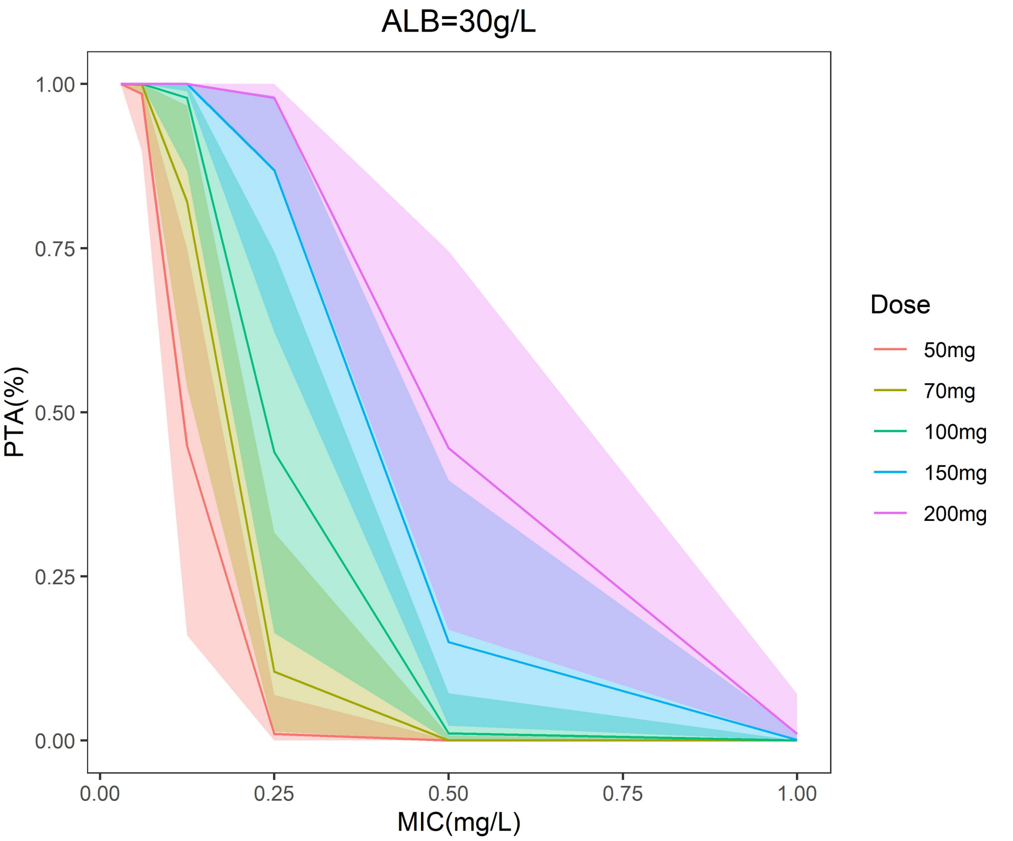
ALB=30g/L
PTA(%)
1.00
0.75
0.50
0.25
0.00
0.00
0.25
0.50
0.75
1.00
MIC(mg/L)
Dose
50mg
70mg
100mg
150mg
200mg

C

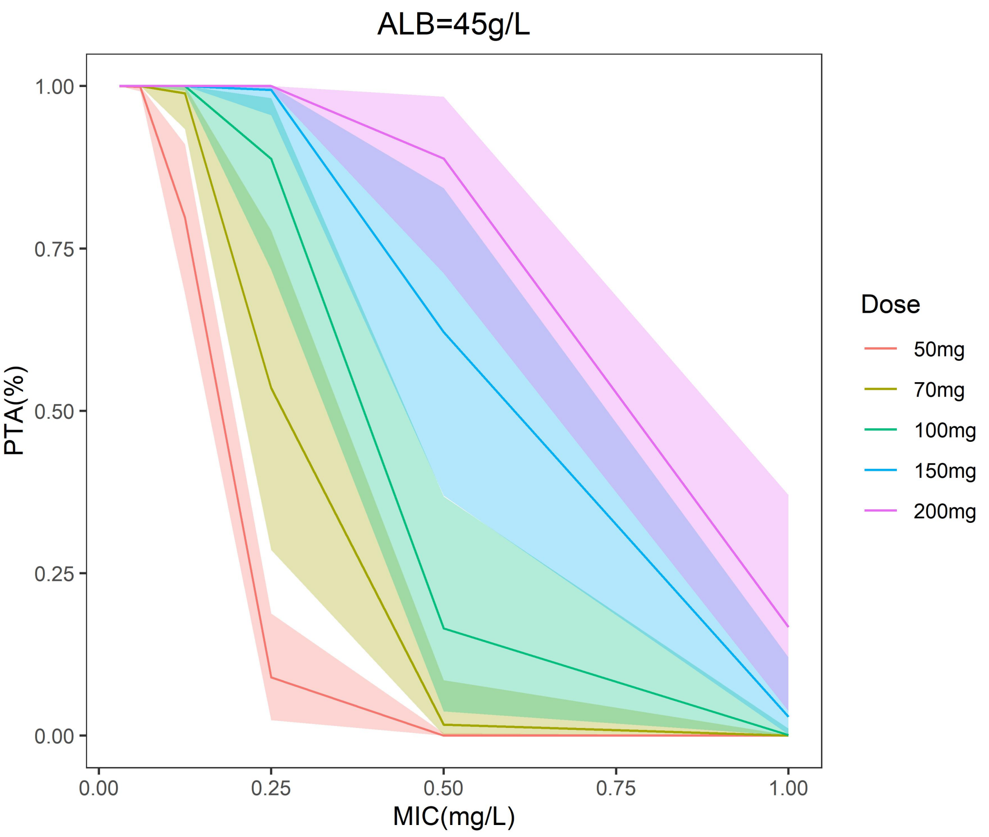

ALB=45g/L
PTA(%)
1.00
0.75
0.50
0.25
0.00
0.00
0.25
0.50
0.75
1.00
MIC(mg/L)
Dose
50mg
70mg
100mg
150mg
200mg

D

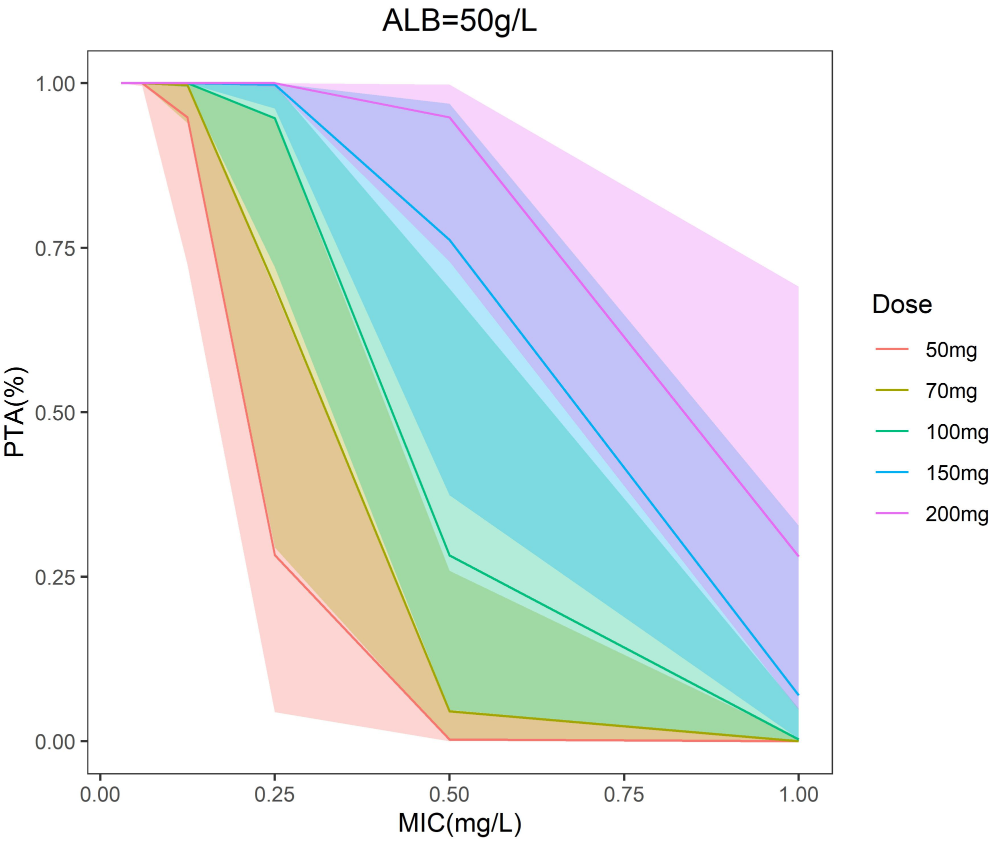

ALB=50g/L
PTA(%)
1.00
0.75
0.50
0.25
0.00
0.00
0.25
0.50
0.75
1.00
MIC(mg/L)
Dose
50mg
70mg
100mg
150mg
200mg

E

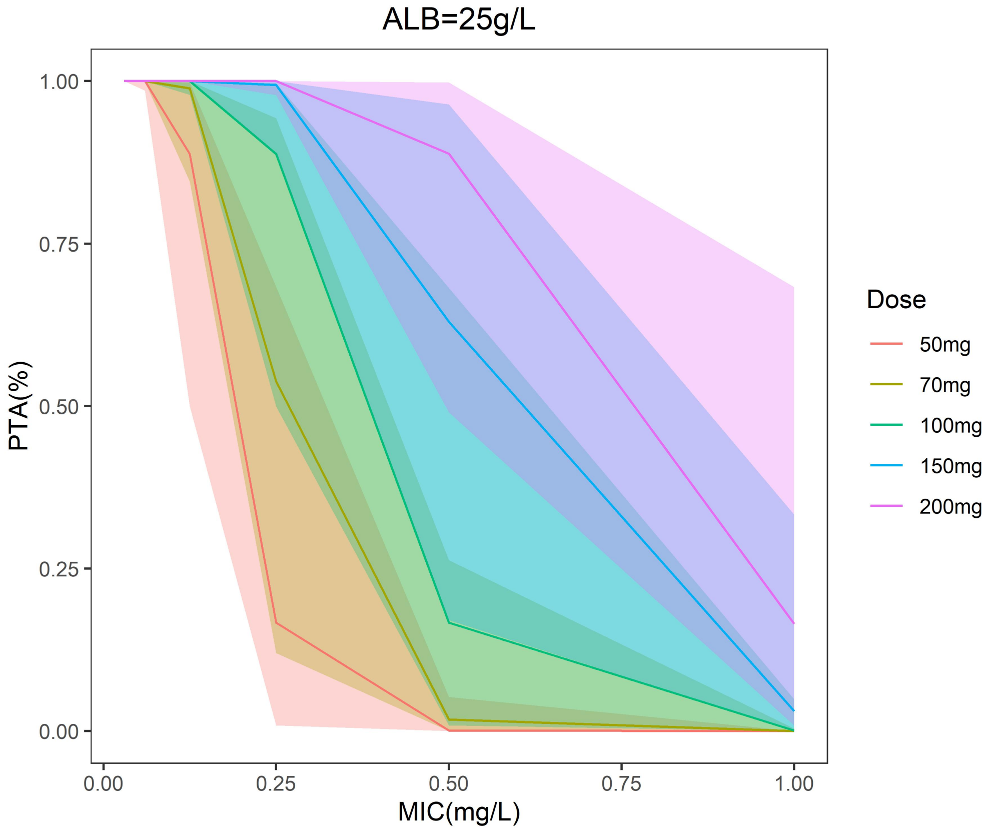
ALB=25g/L
1.00
0.75
0.50
0.25
0.00
PTA(%)
0.00
0.25
0.50
0.75
1.00
MIC(mg/L)
Dose
50mg
70mg
100mg
150mg
200mg

F

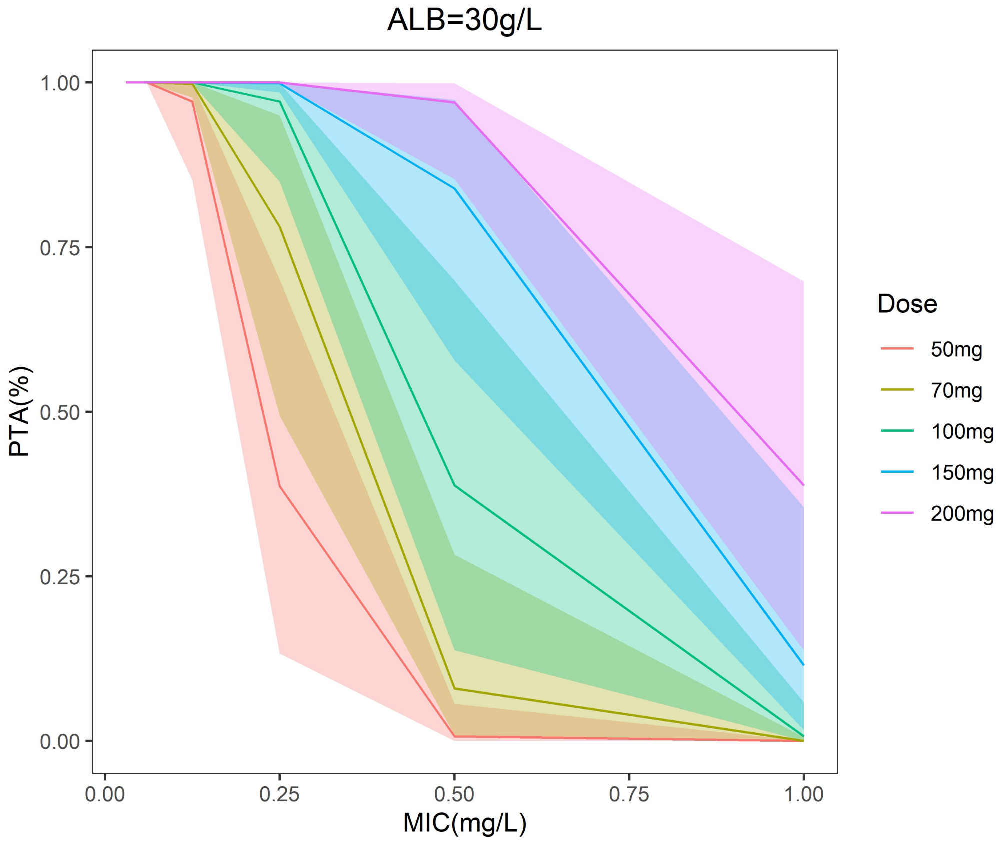
ALB=30g/L
1.00
0.75
0.50
0.25
0.00
PTA(%)
0.00
0.25
0.50
0.75
1.00
MIC(mg/L)
Dose
50mg
70mg
100mg
150mg
200mg

G

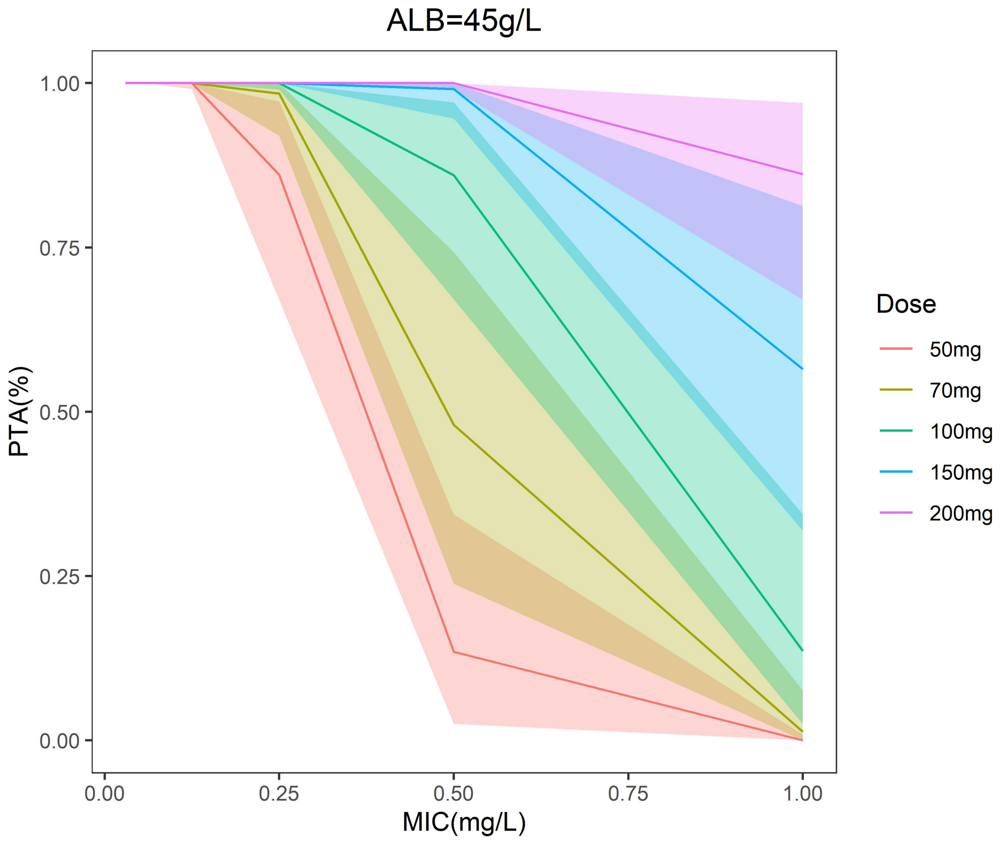
ALB=45g/L
1.00
0.75
0.50
0.25
0.00
PTA(%)
0.00
0.25
0.50
0.75
1.00
MIC(mg/L)
Dose
50mg
70mg
100mg
150mg
200mg

H

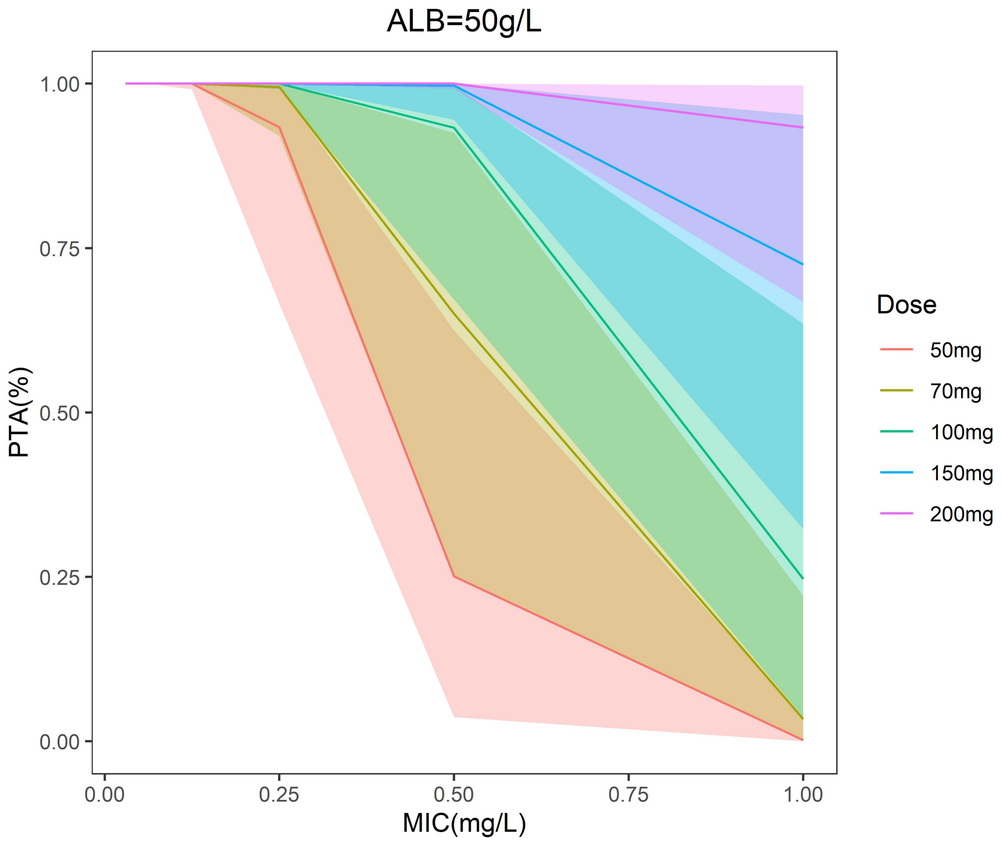
ALB=50g/L
1.00
0.75
0.50
0.25
0.00
PTA(%)
0.00
0.25
0.50
0.75
1.00
MIC(mg/L)
Dose
50mg
70mg
100mg
150mg
200mg

I

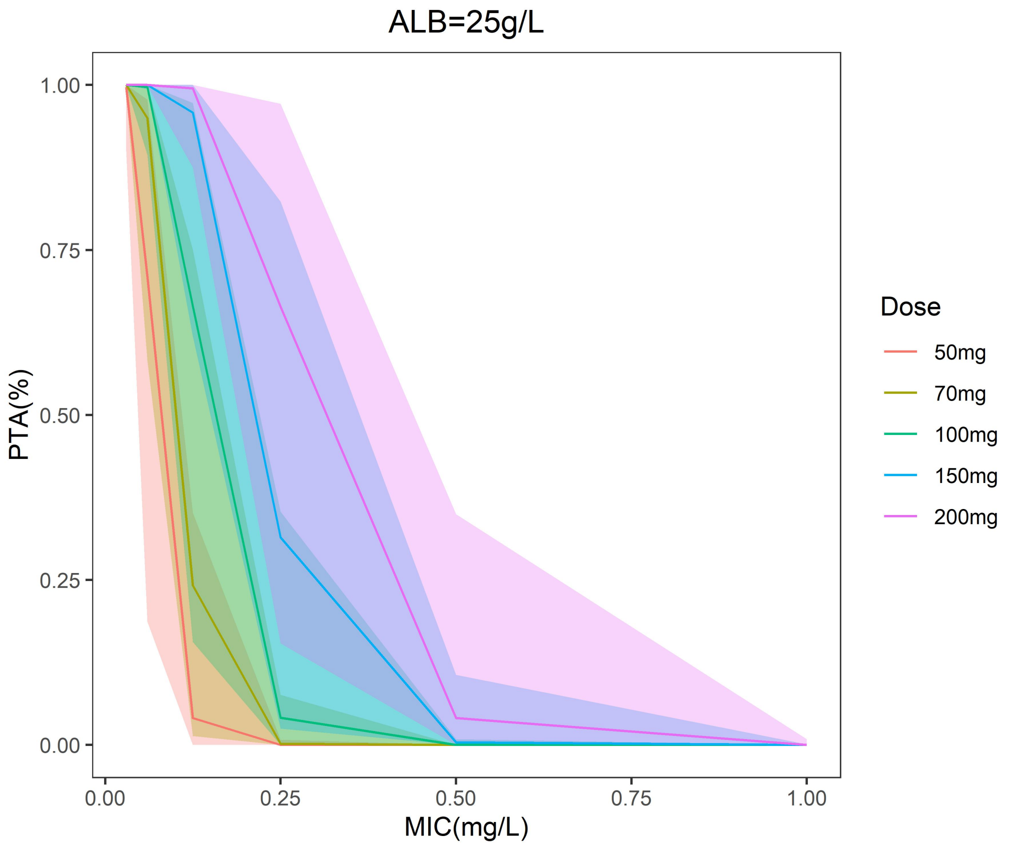

ALB=25g/L
PTA(%)
1.00
0.75
0.50
0.25
0.00
0.00
0.25
0.50
0.75
1.00
MIC(mg/L)
Dose
50mg
70mg
100mg
150mg
200mg

J

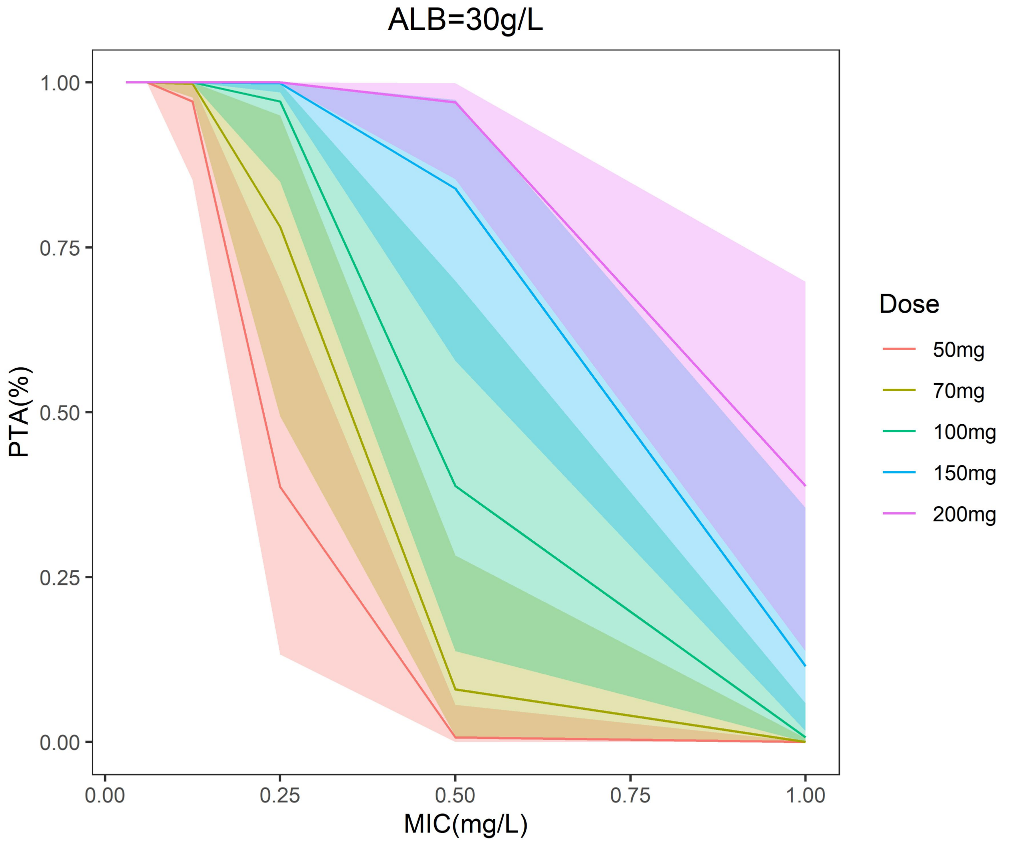

ALB=30g/L
PTA(%)
1.00
0.75
0.50
0.25
0.00
0.00
0.25
0.50
0.75
1.00
MIC(mg/L)
Dose
50mg
70mg
100mg
150mg
200mg

K

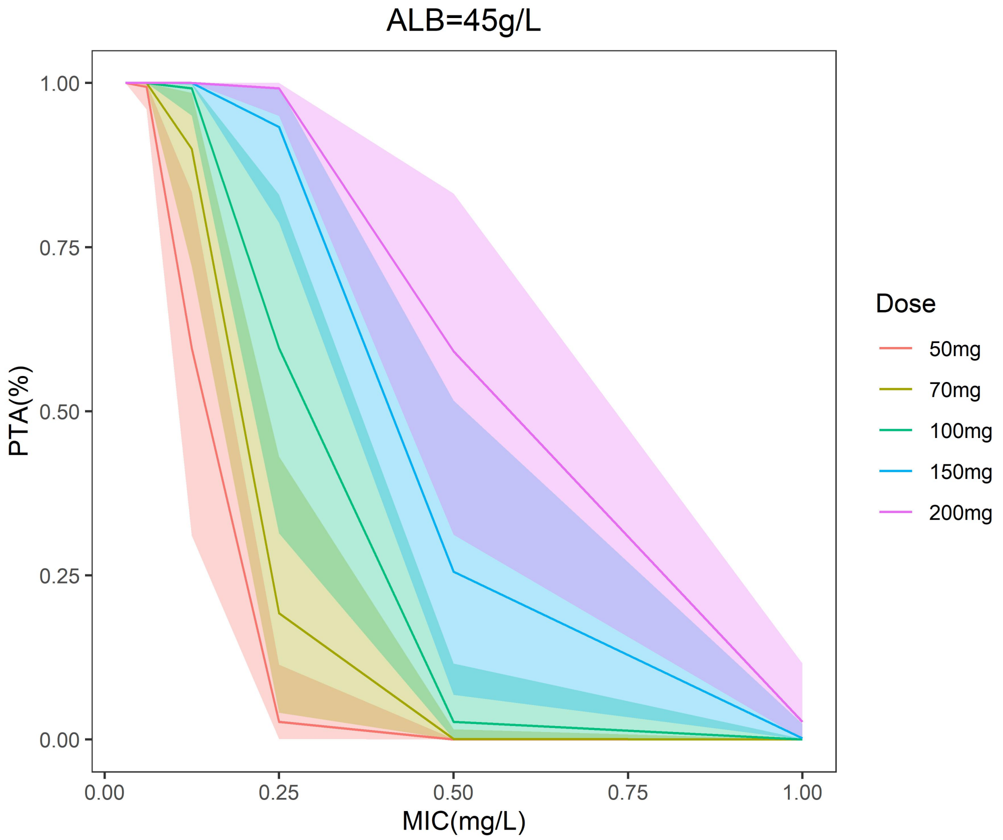
ALB=45g/L
1.00
0.75
0.50
0.25
0.00
PTA(%)
0.00
0.25
0.50
0.75
1.00
MIC(mg/L)
Dose
50mg
70mg
100mg
150mg
200mg

L

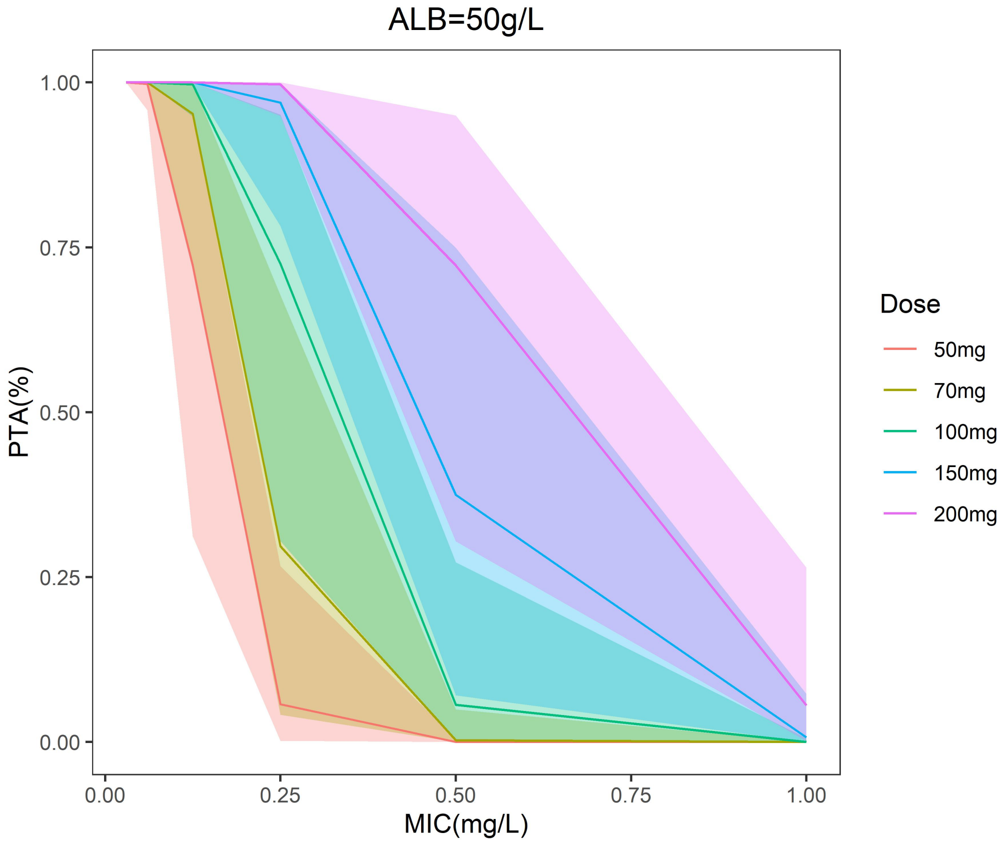
ALB=50g/L
1.00
0.75
0.50
0.25
0.00
PTA(%)
0.00
0.25
0.50
0.75
1.00
MIC(mg/L)
Dose
50mg
70mg
100mg
150mg
200mg

Figure S1 Probability of target attainment (PTA) of caspofungin versus *Candida.albicans* (A-D), *Candida.glabrata* (E-H) and *Candida. parapsilosis* (I-L). The shade around the lines represents the 95% confidence intervals of the prediction.

A

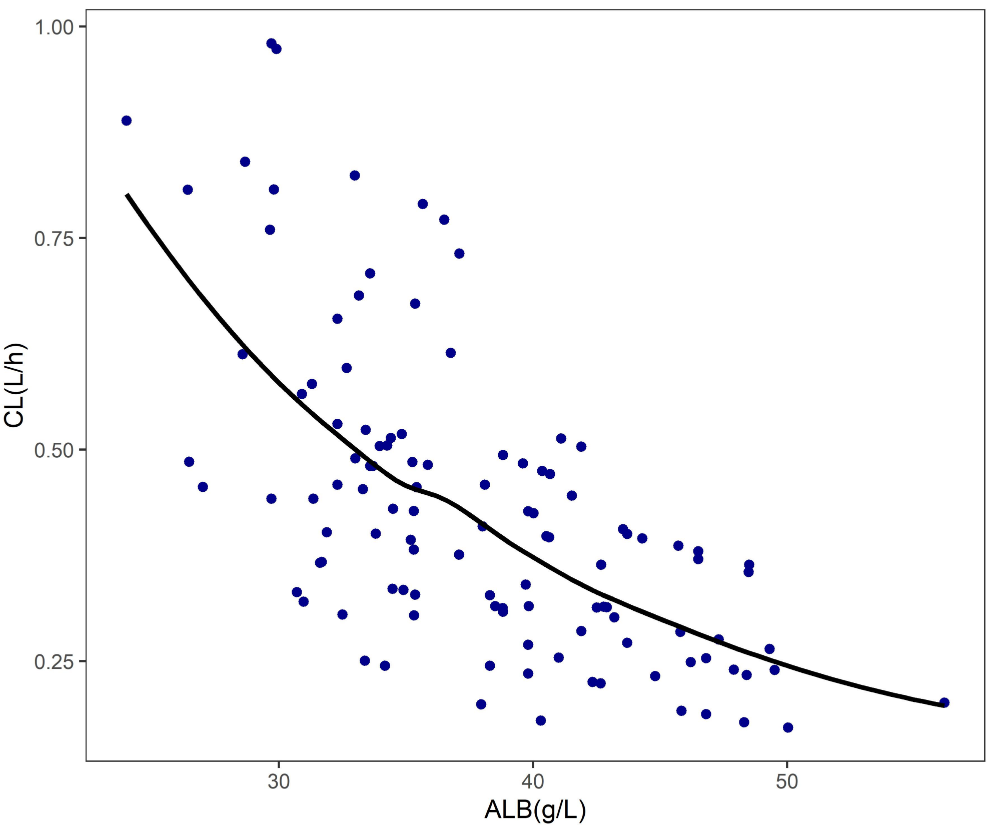

1.00
0.75
0.50
0.25
CL(L/h)
30
40
50
ALB(g/L)

B

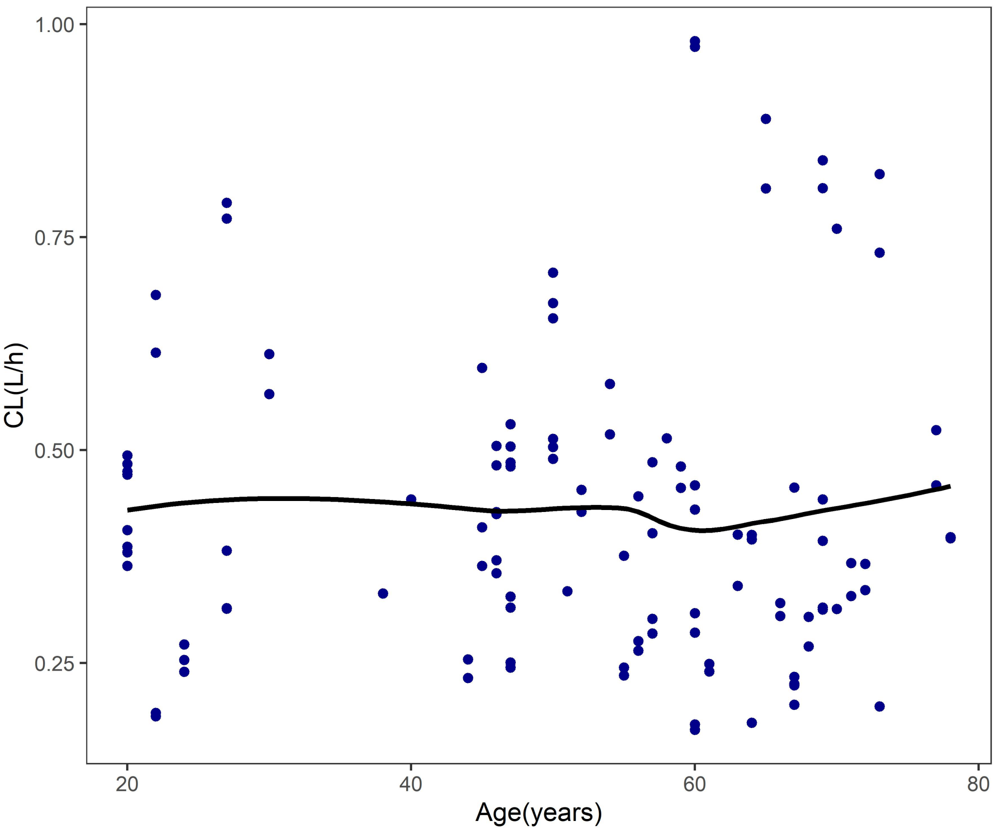

1.00
0.75
0.50
0.25
CL(L/h)
20
40
60
80
Age(years)

C

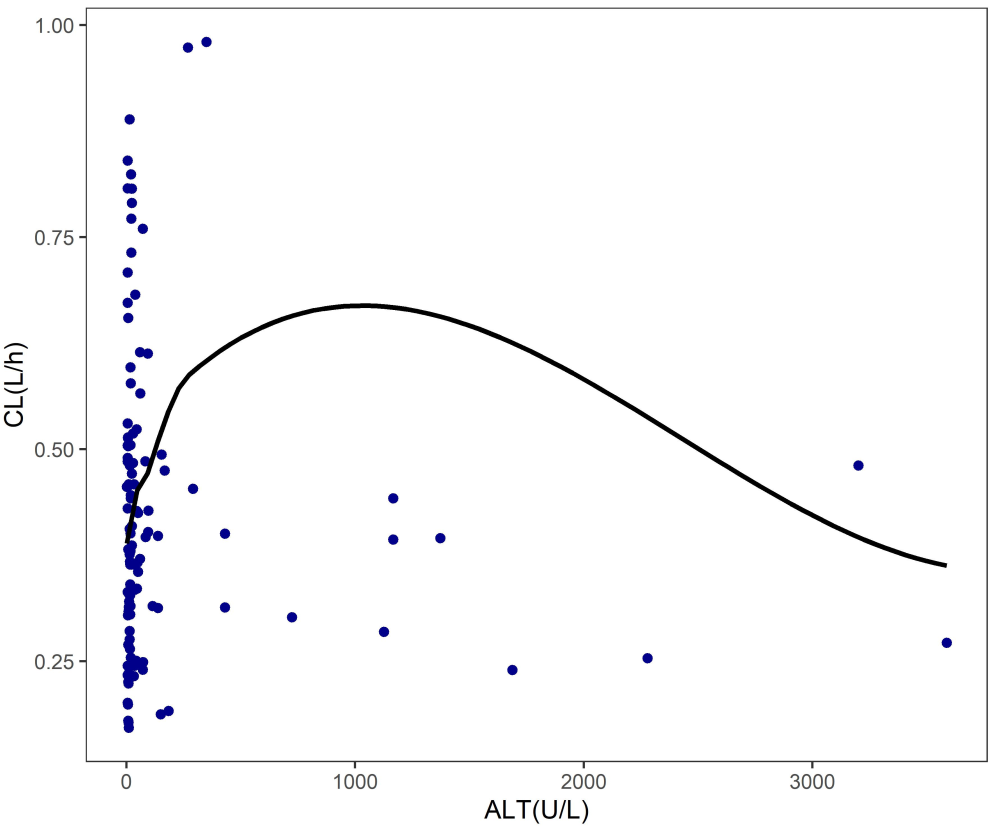
1.00
0.75
0.50
0.25
CL(L/h)
0
1000
2000
3000
ALT(U/L)

D

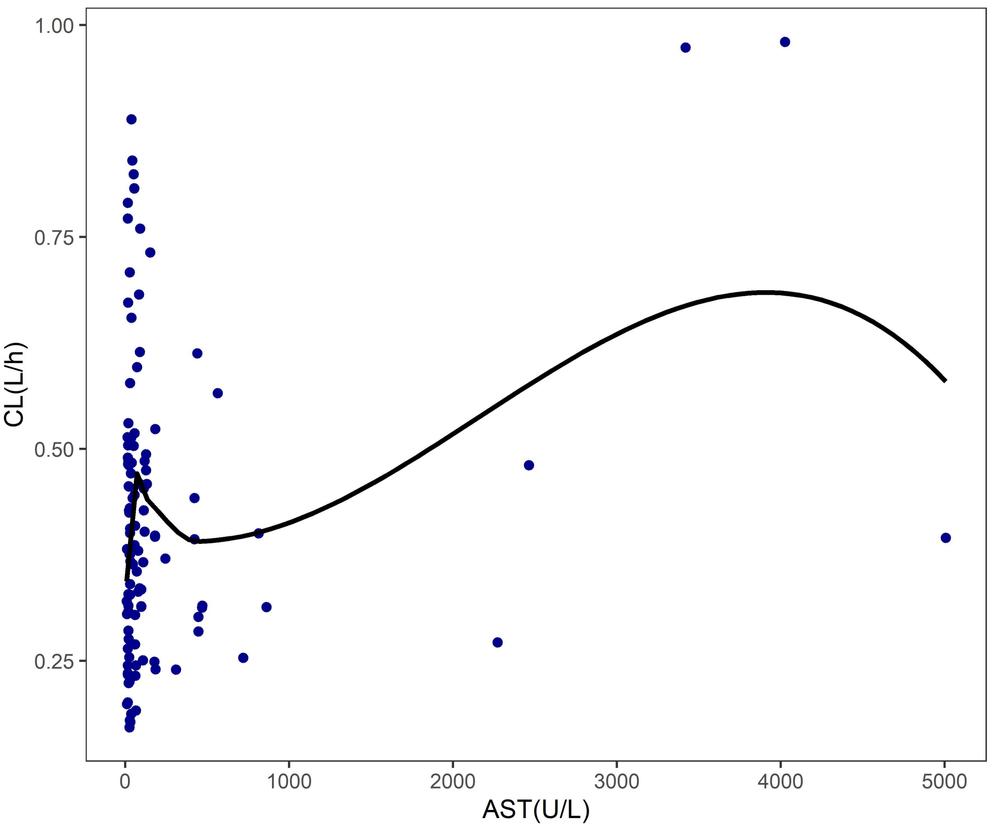
1.00
0.75
0.50
0.25
CL(L/h)
0
1000
2000
3000
4000
5000
AST(U/L)

E

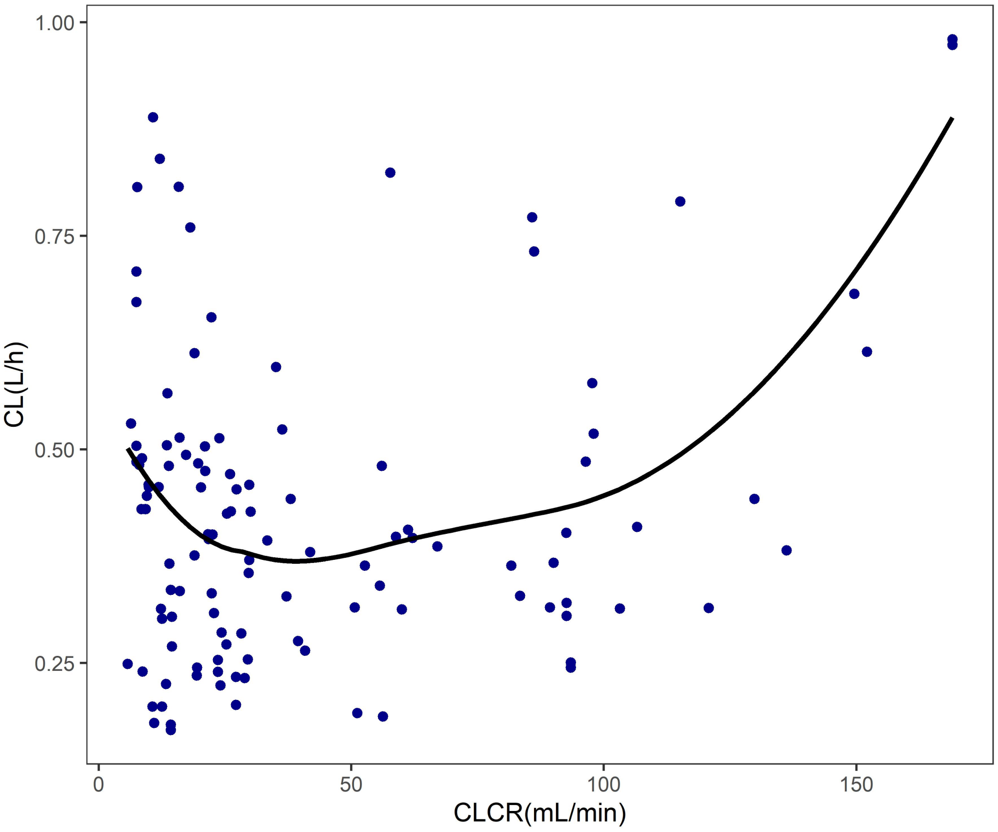
1.00
0.75
0.50
0.25
CL(L/h)
0
50
100
150
CLCR(mL/min)

F

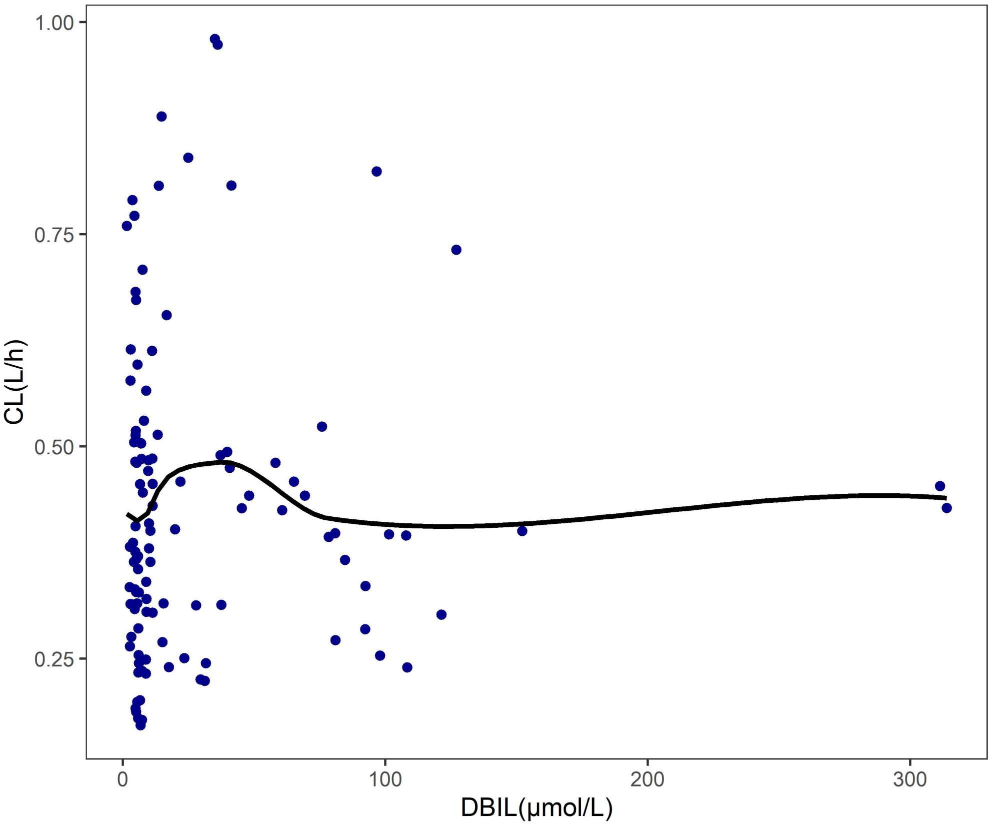
1.00
0.75
0.50
0.25
CL(L/h)
0
100
200
300
DBIL(μmol/L)

G

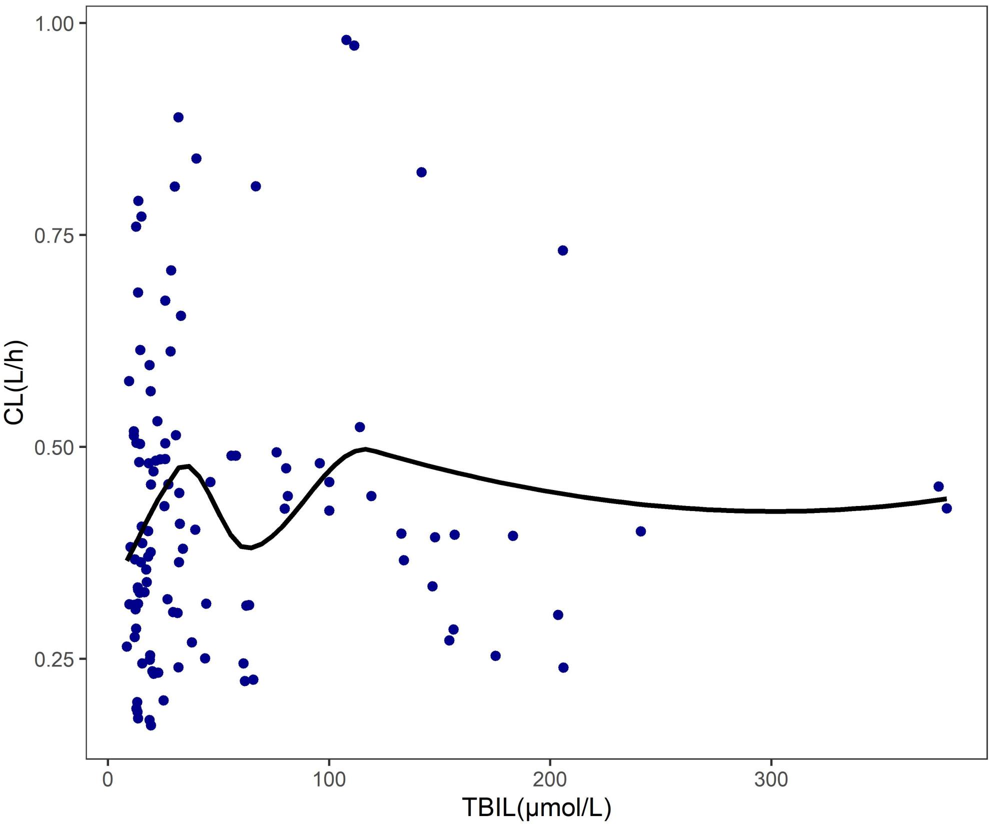
1.00
0.75
0.50
0.25
CL(L/h)
0
100
200
300
TBIL(μmol/L)

H

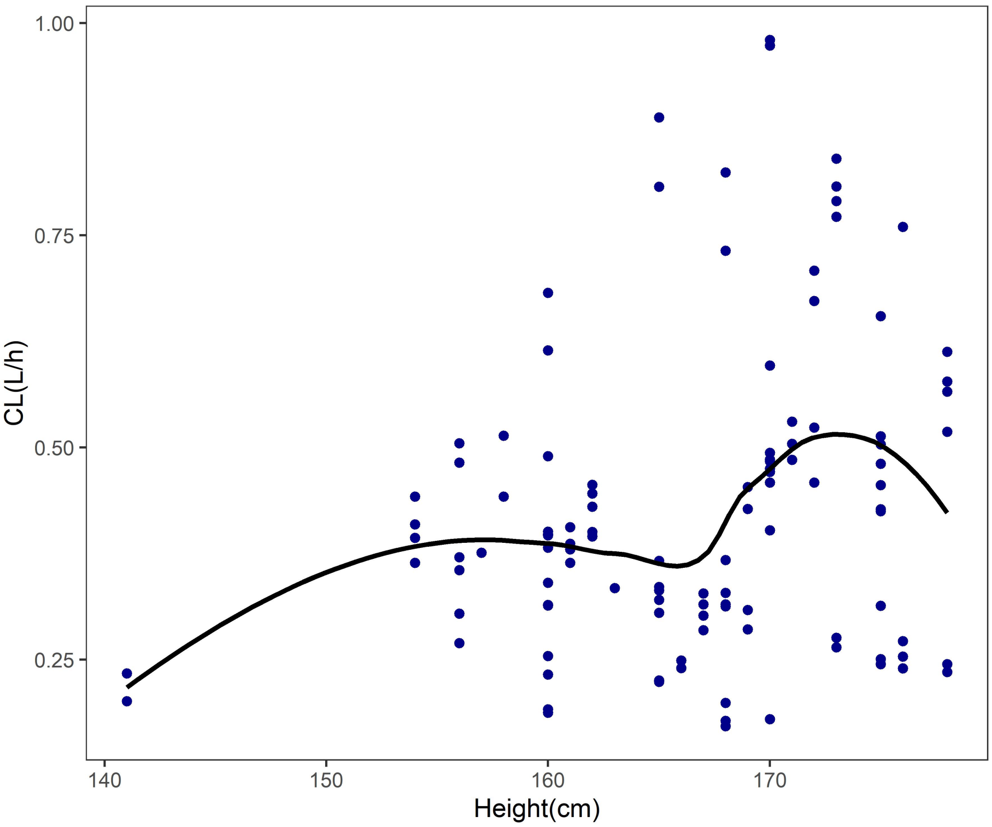
1.00
0.75
0.50
0.25
CL(L/h)
140
150
160
170
Height(cm)

I

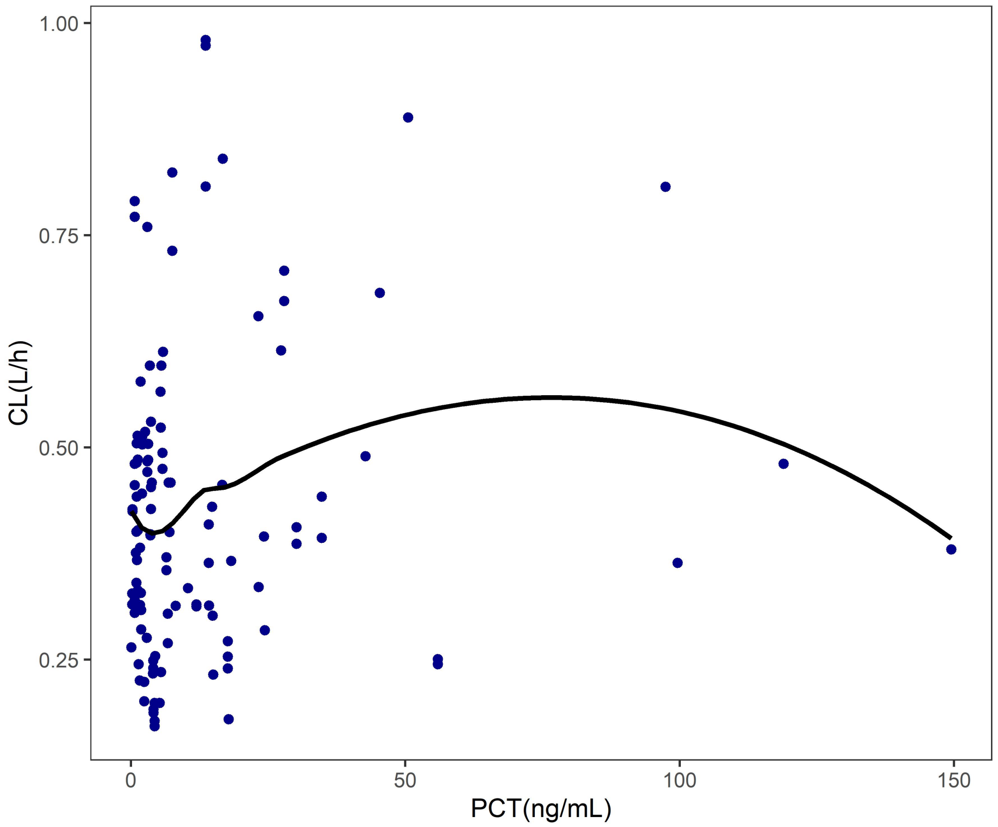

1.00
0.75
0.50
0.25
CL(L/h)
0
50
100
150
PCT(ng/mL)

J

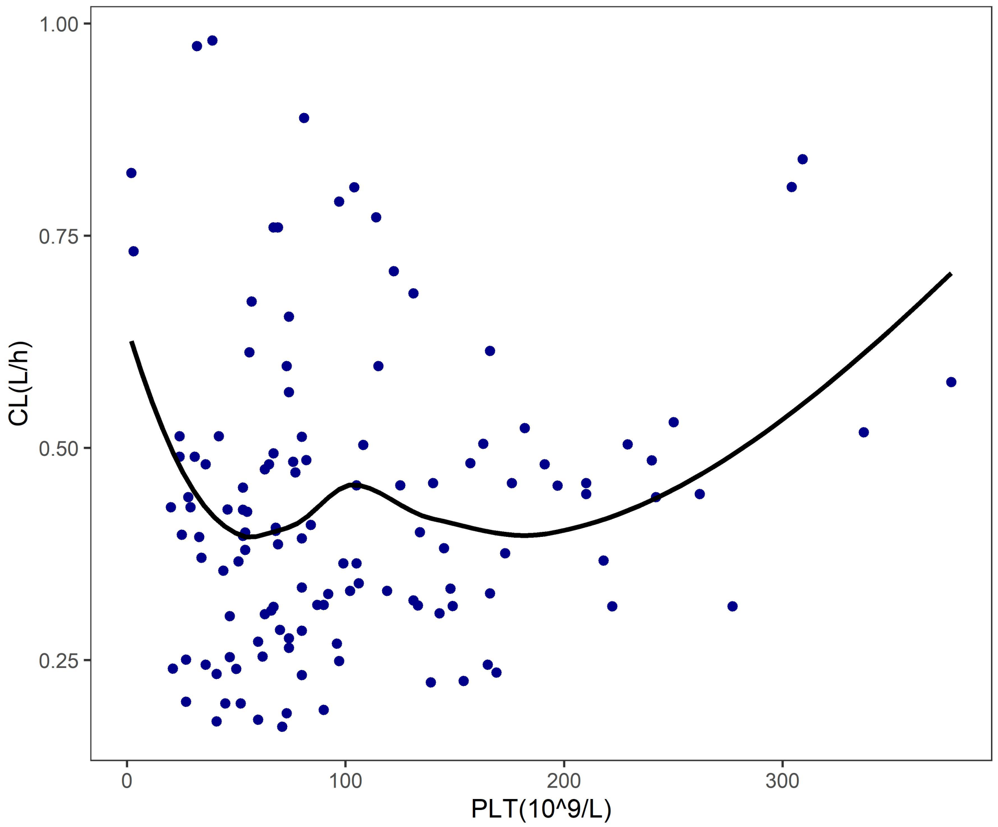

1.00
0.75
0.50
0.25
CL(L/h)
0
100
200
300
PLT(10^9/L)

K

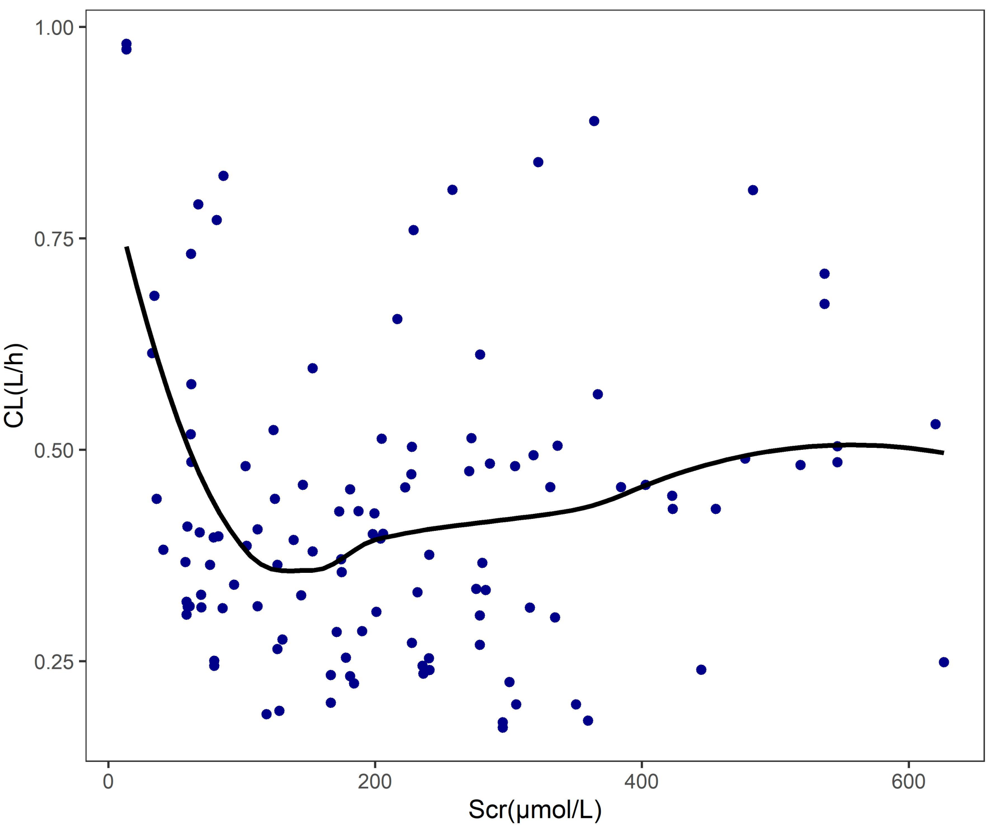

CL(L/h)
1.00
0.75
0.50
0.25
0
200
400
600
Scr(μmol/L)

L

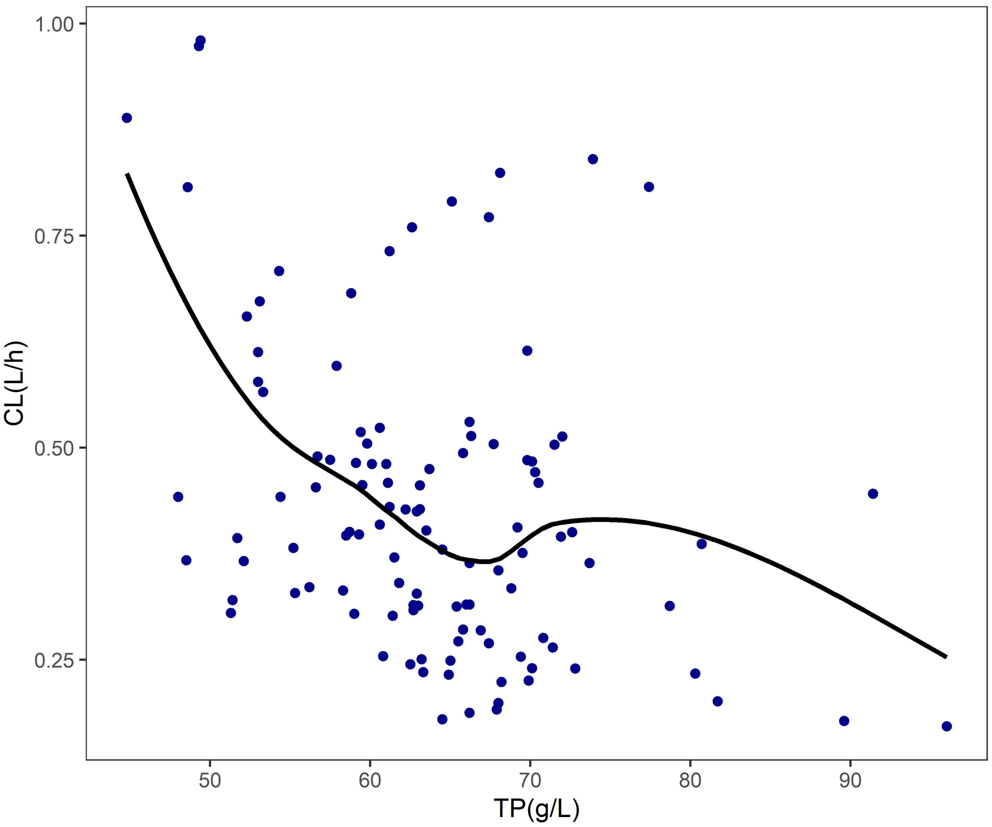

CL(L/h)
1.00
0.75
0.50
0.25
50
60
70
80
90
TP(g/L)

M

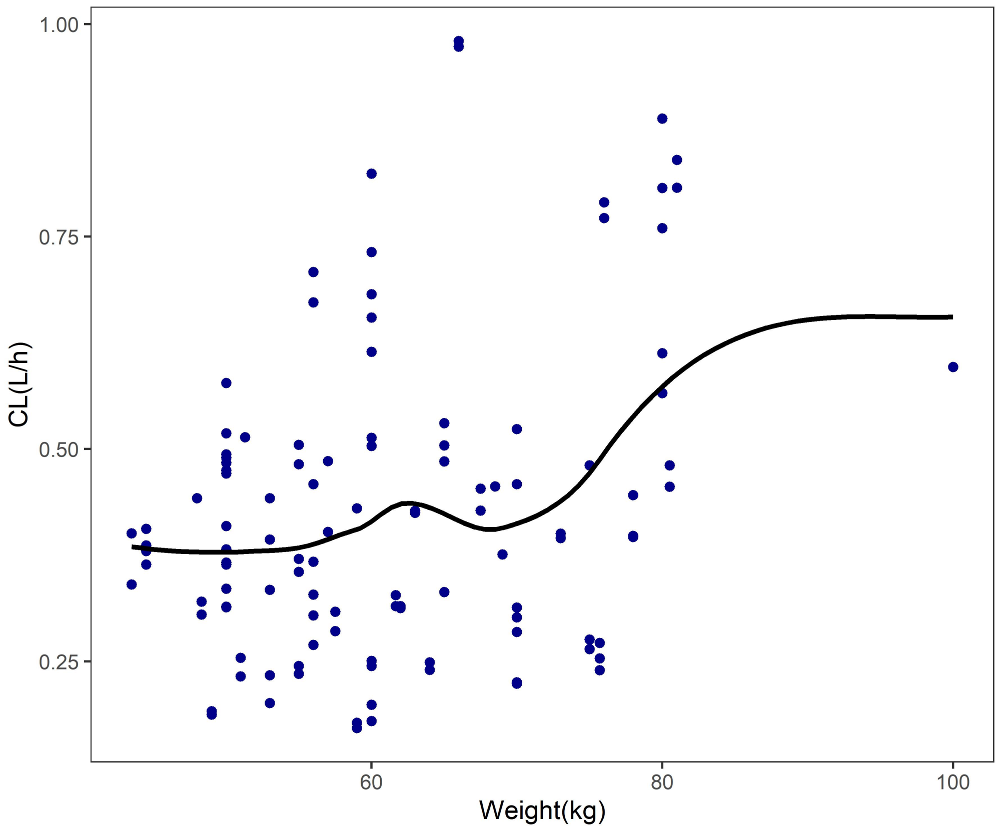
1.00
0.75
0.50
0.25
CL(L/h)
60
80
100
Weight(kg)

N

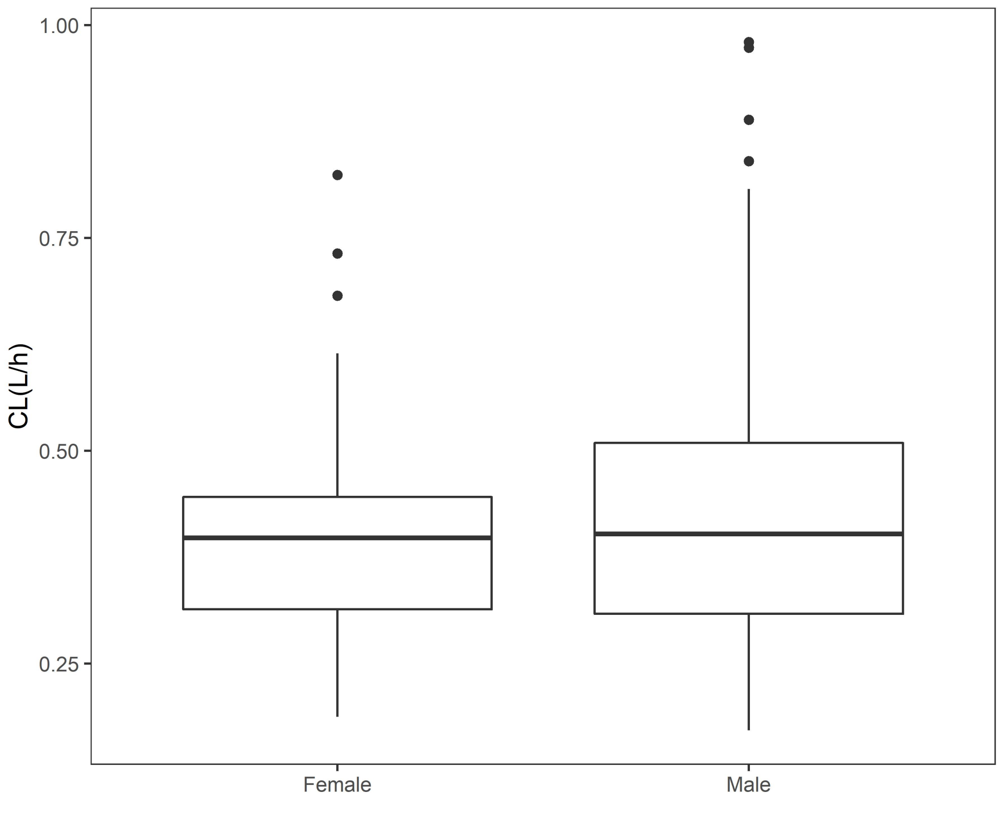
1.00
0.75
0.50
0.25
CL(L/h)
Female
Male

O

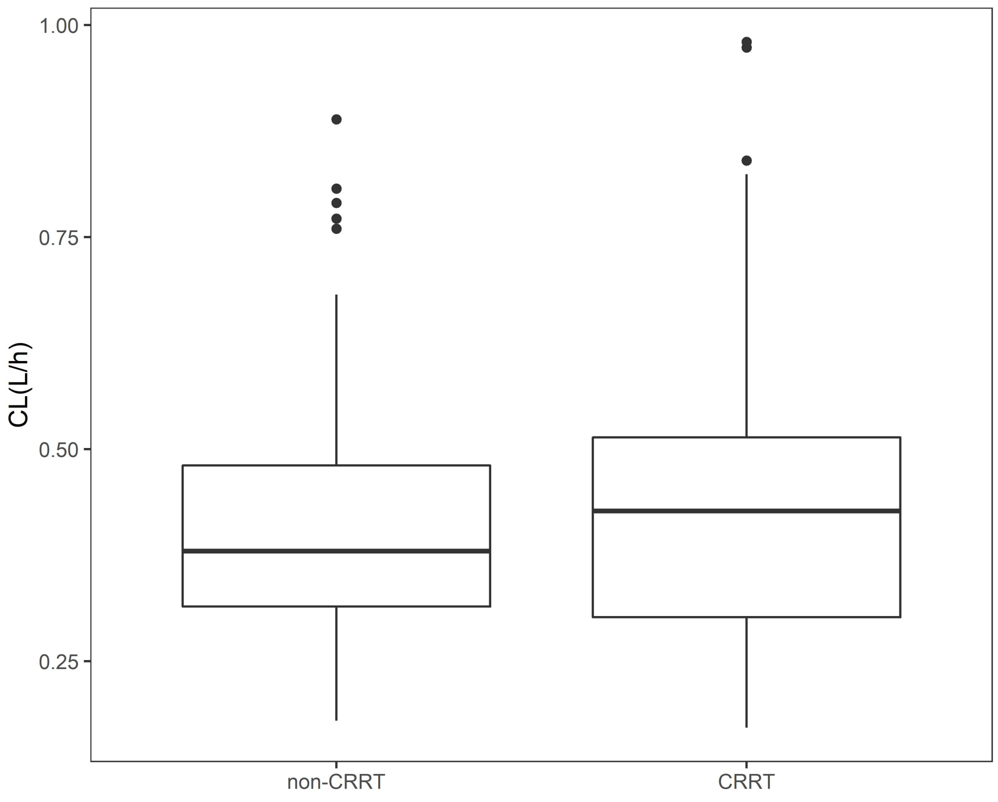
1.00
0.75
0.50
0.25
CL(L/h)
non-CRRT
CRRT

P

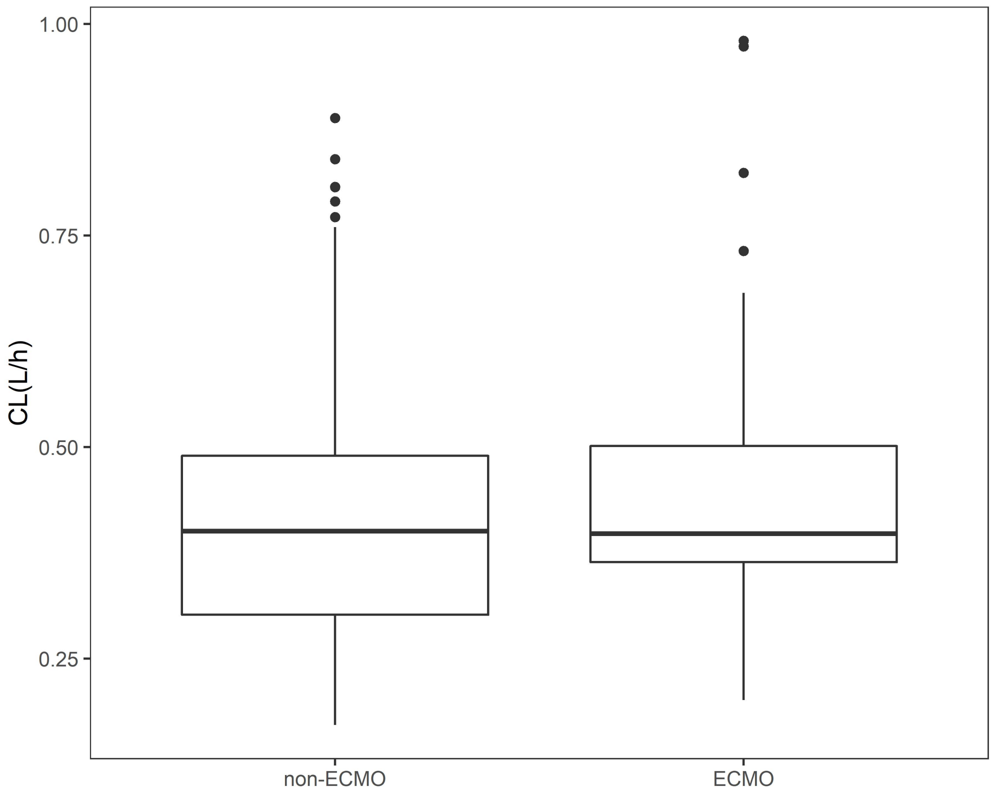
1.00
0.75
0.50
0.25
CL(L/h)
non-ECMO
ECMO

Q

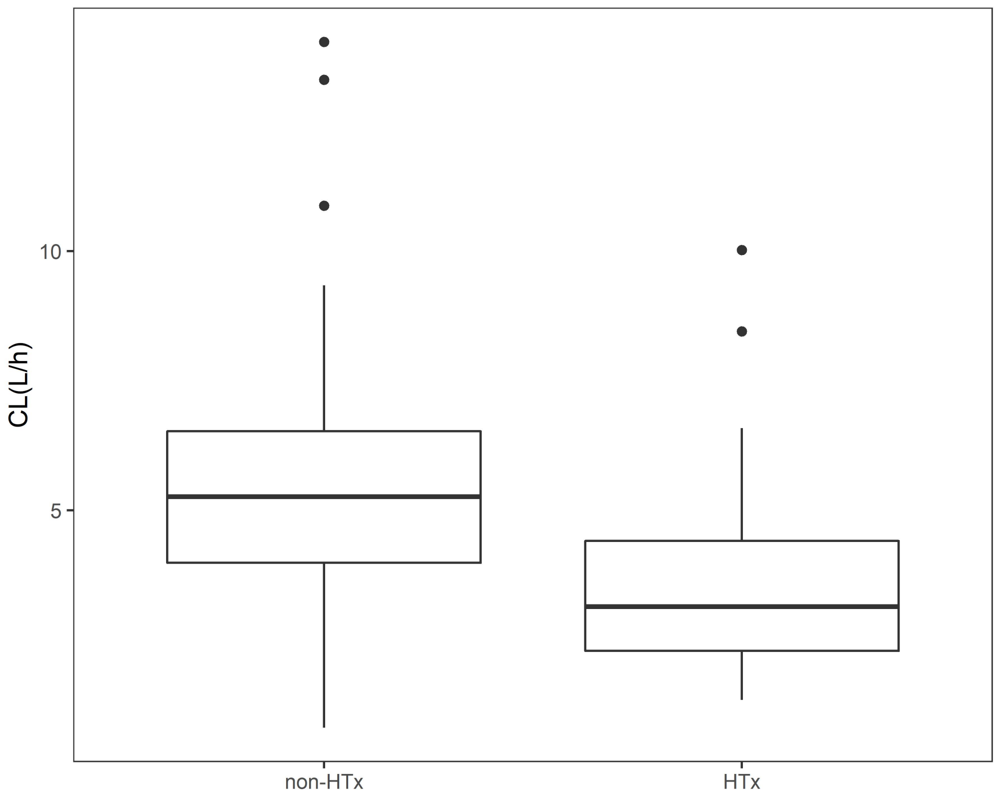

A1

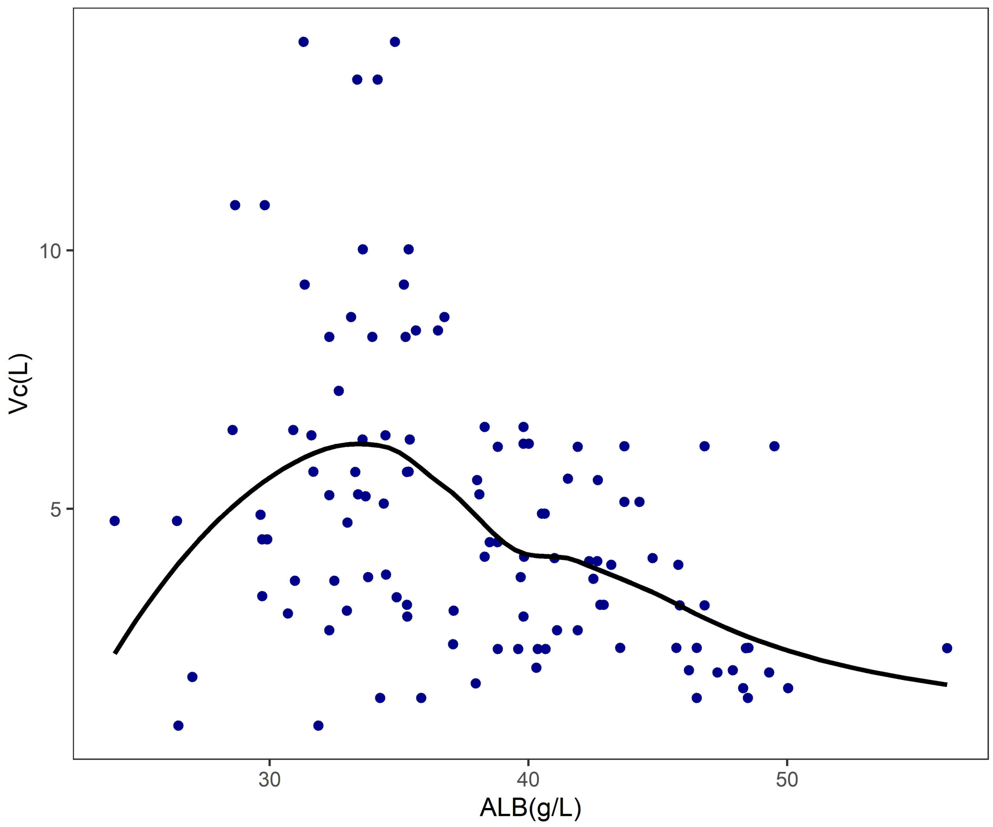

B1

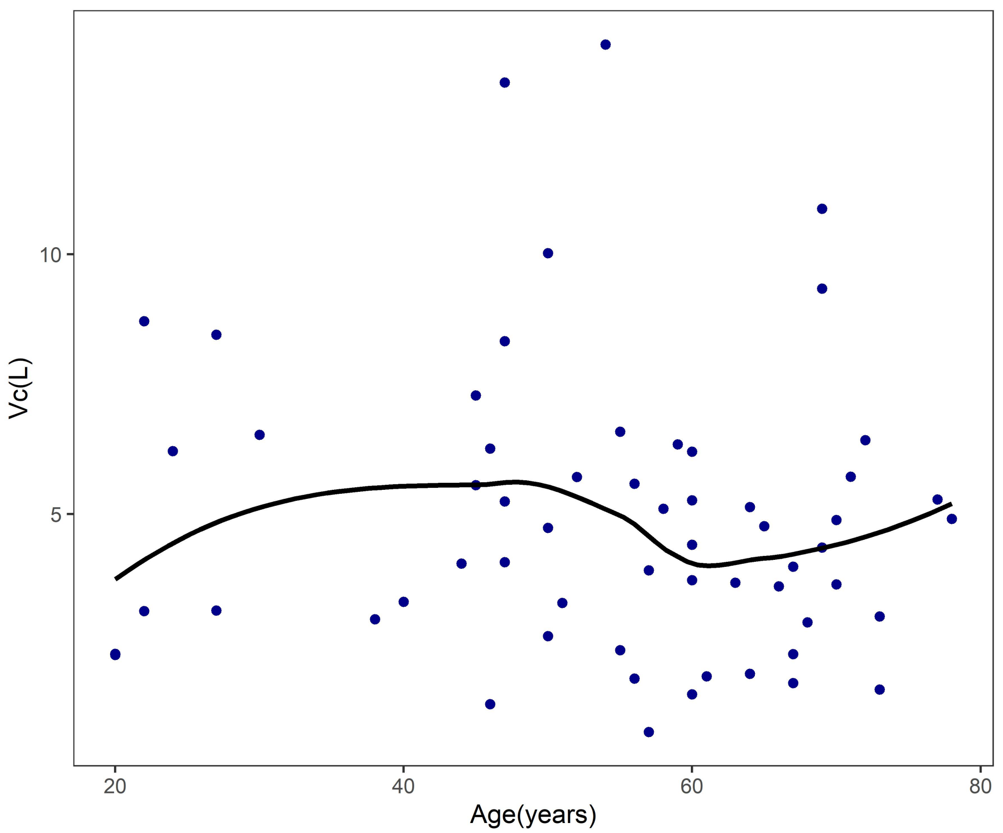

C1

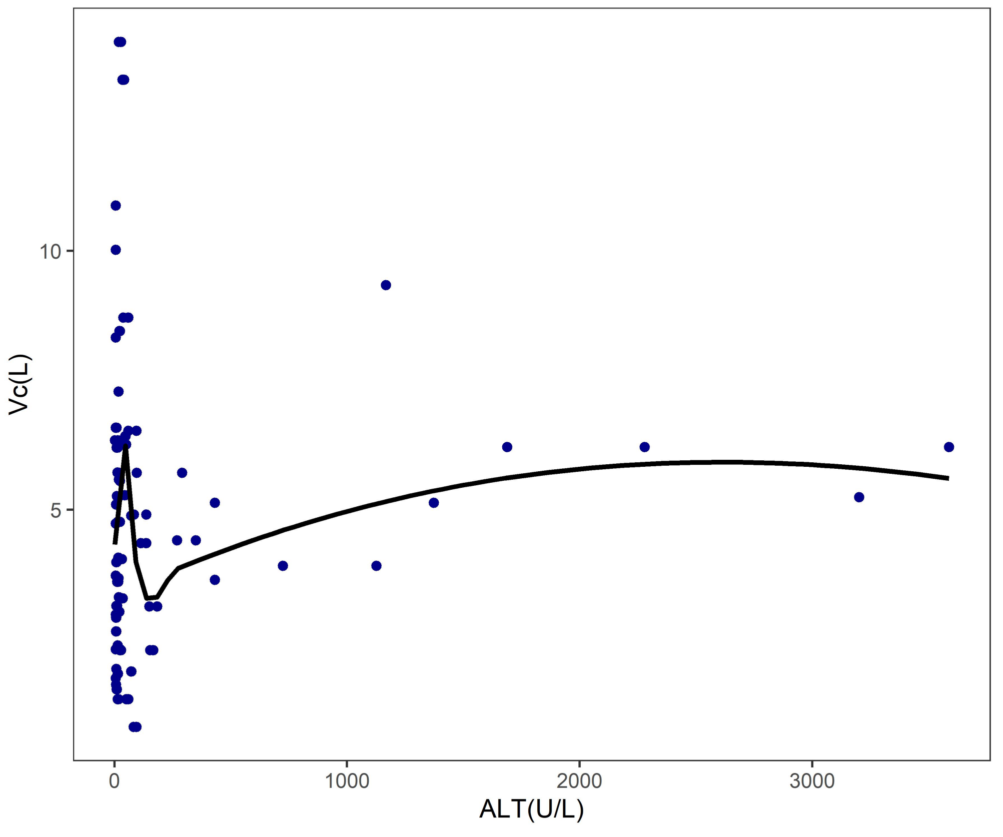

D1

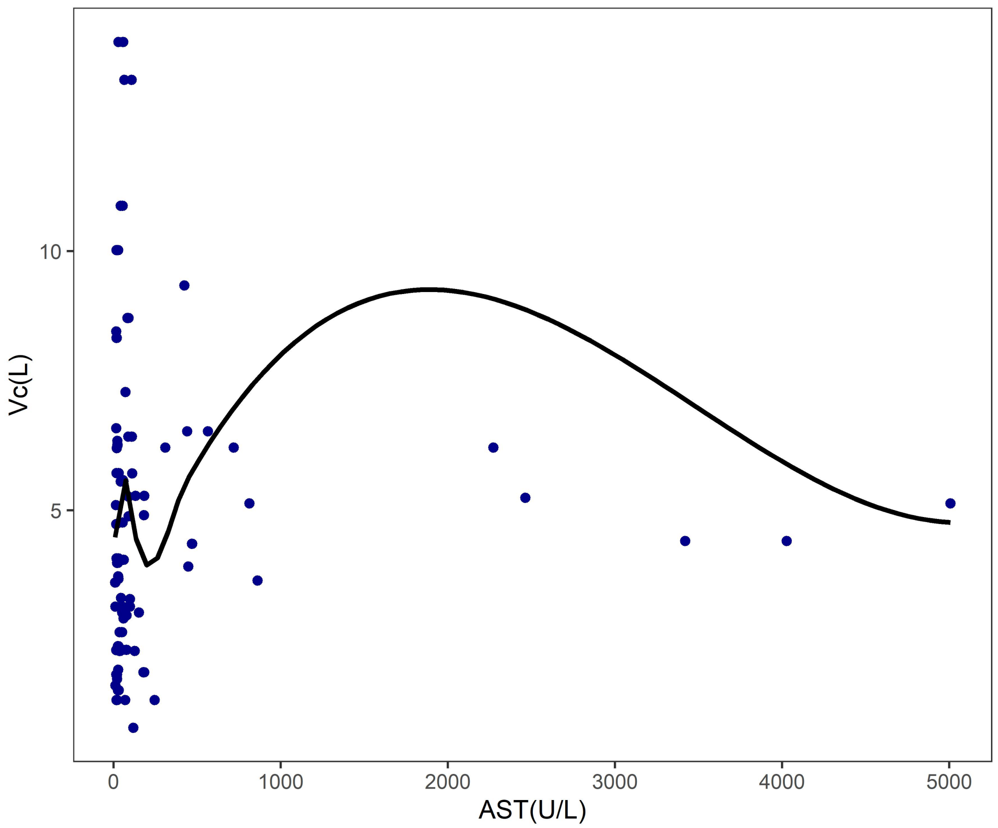

Vc(L)
10
5
0
1000
2000
3000
4000
5000
AST(U/L)

E1

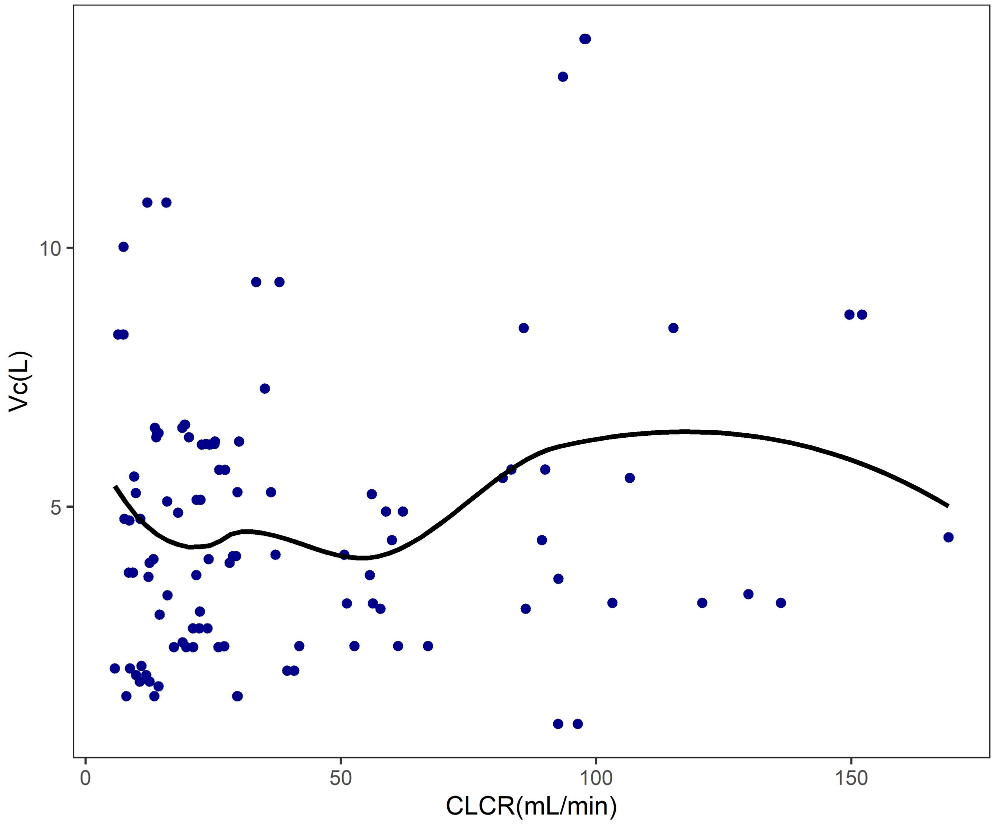

Vc(L)
10
5
0
50
100
150
CLCR(mL/min)

F1

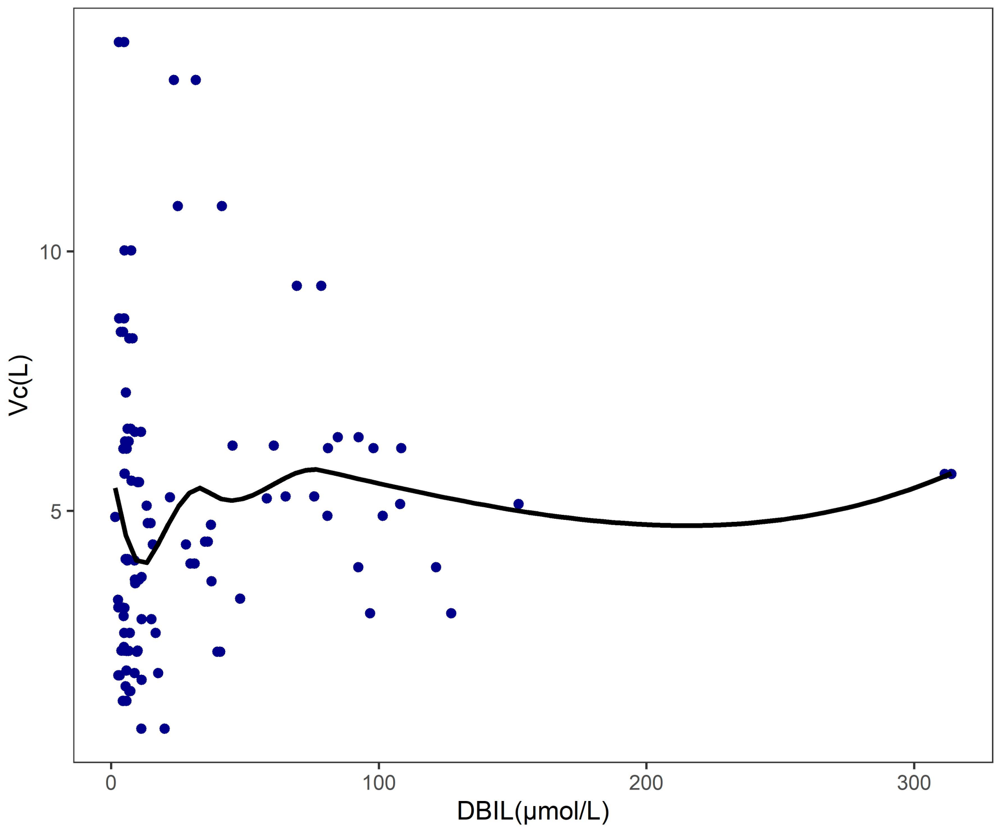
Vc(L)
10
5
0
100
200
300
DBIL(μmol/L)

G1

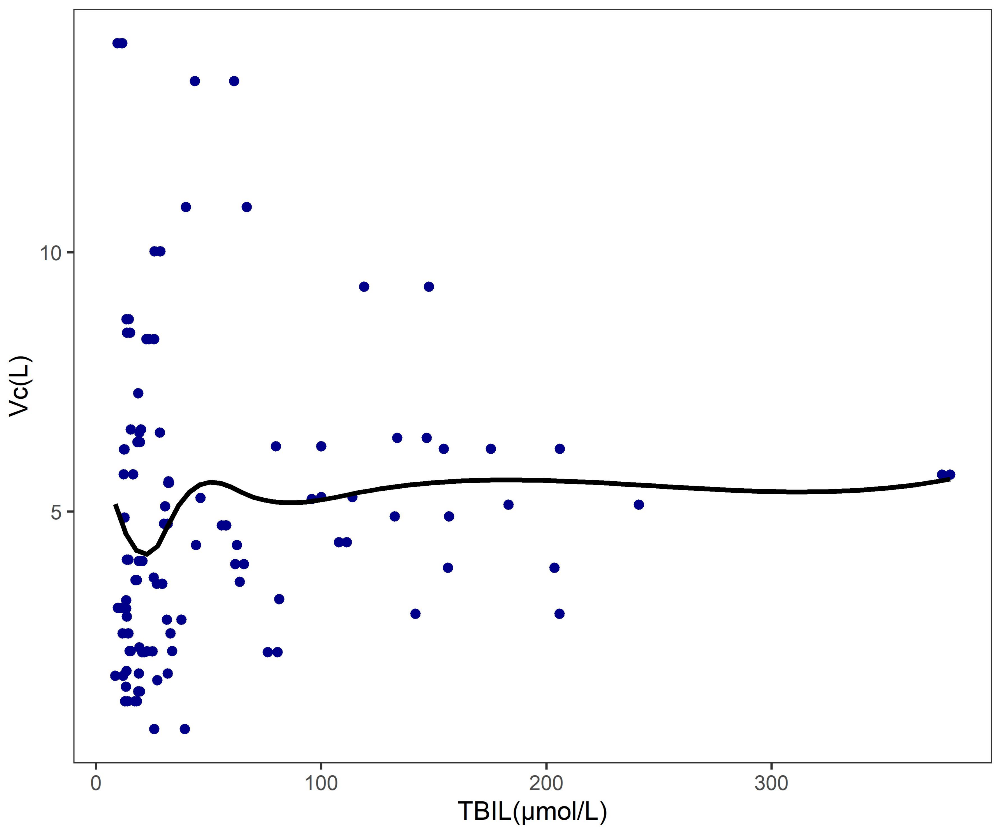
Vc(L)
10
5
0
100
200
300
TBIL(μmol/L)

H1

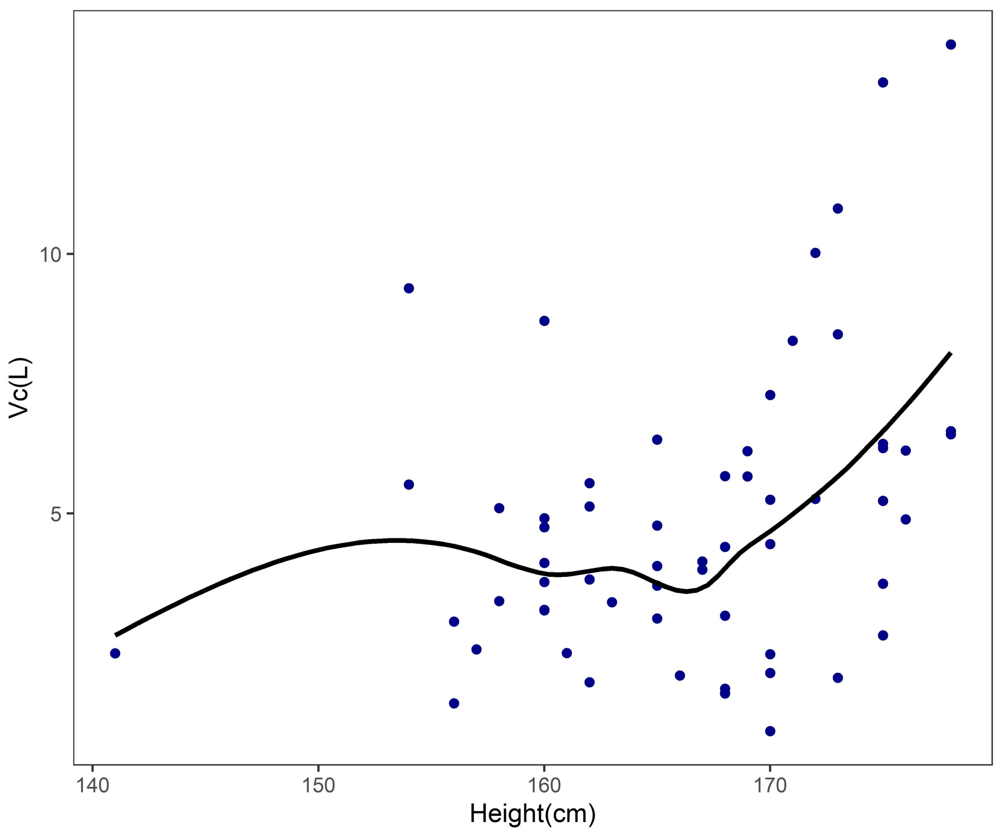

I1

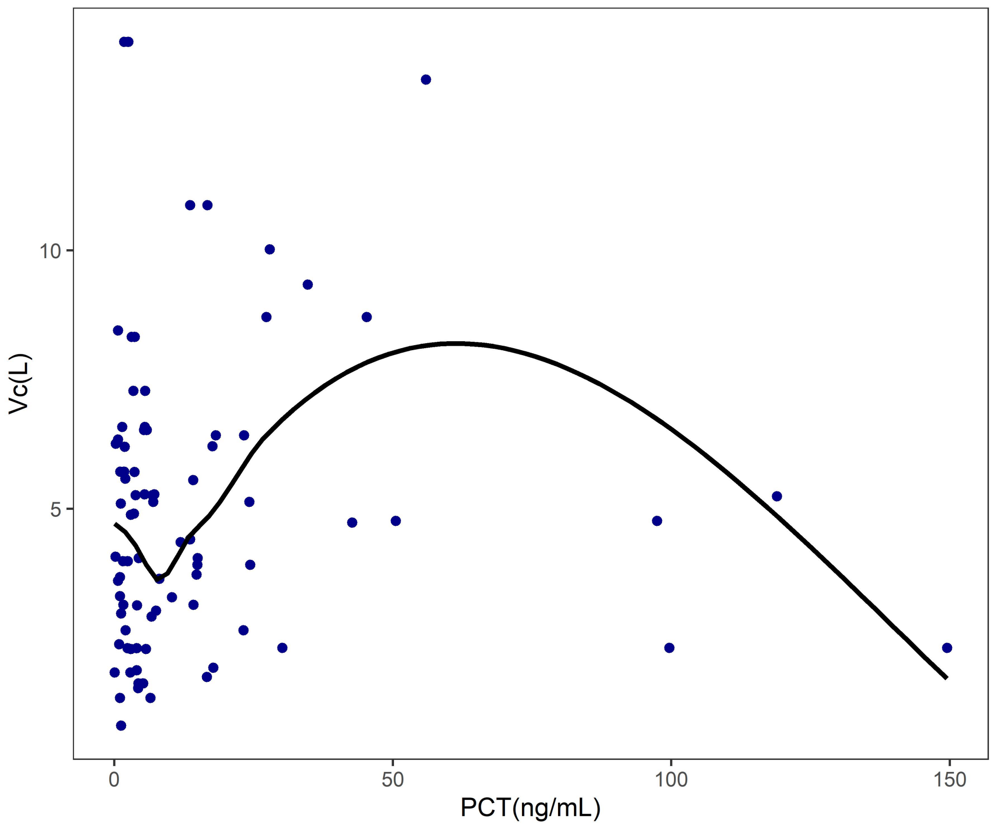

J1

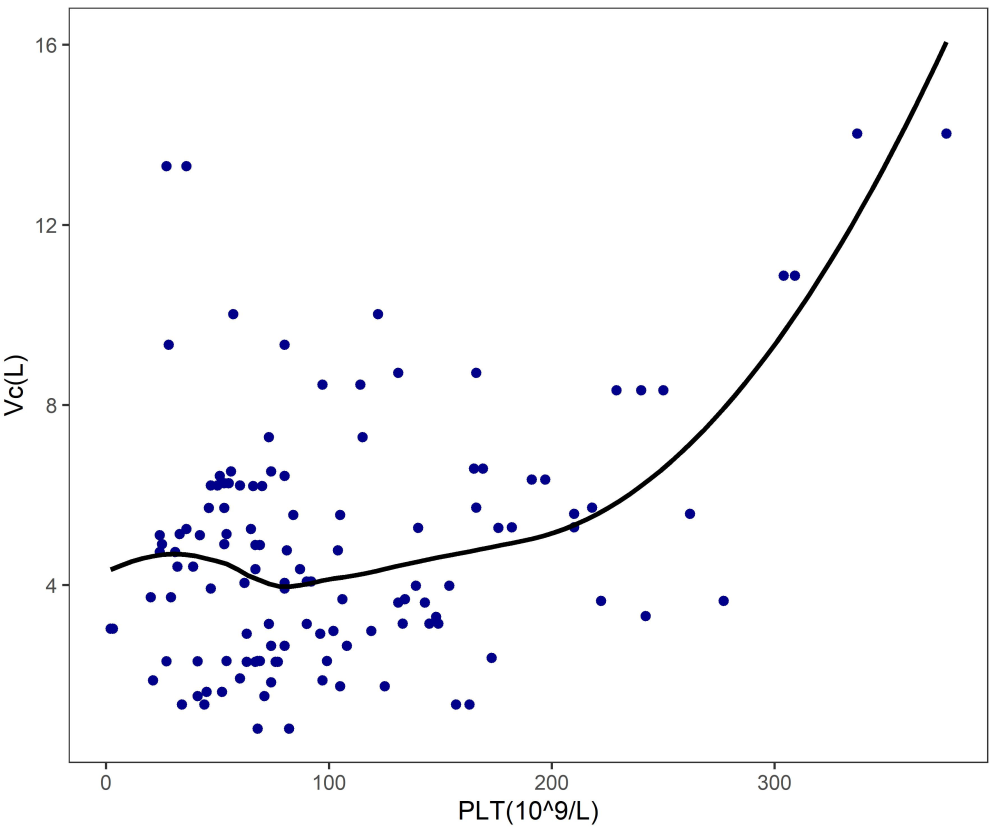
16
12
8
4
Vc(L)
0
100
200
300
PLT(10^9/L)

K1

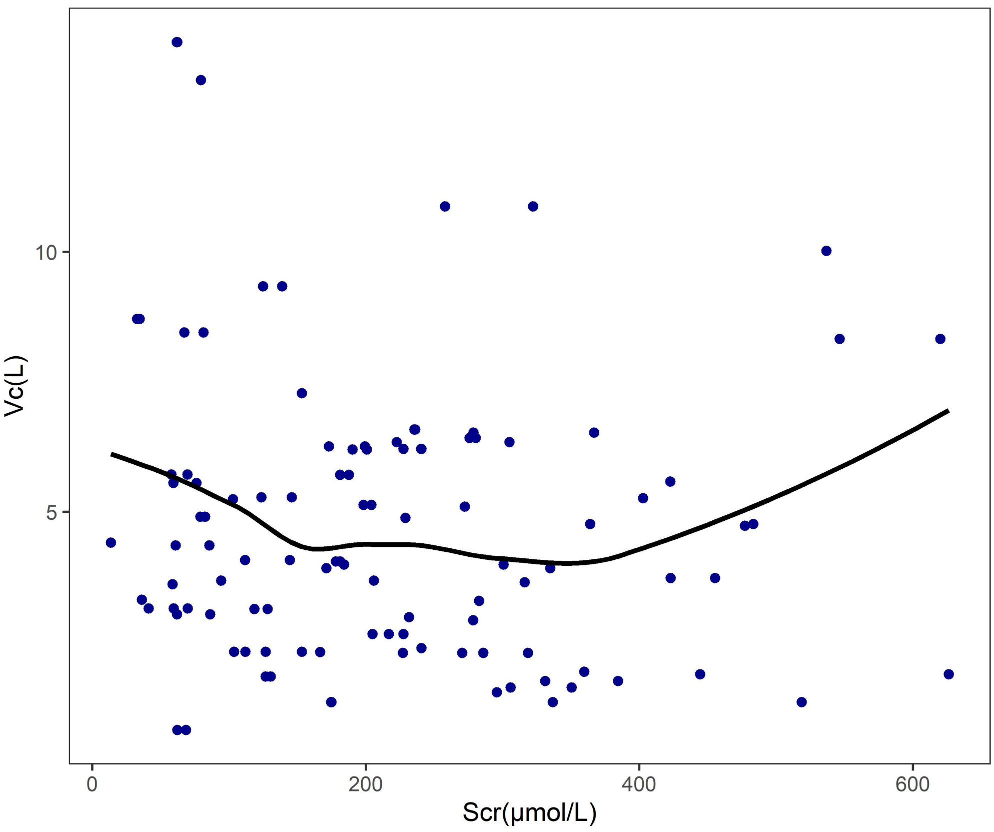
10
5
Vc(L)
0
200
400
600
Scr(μmol/L)

L1

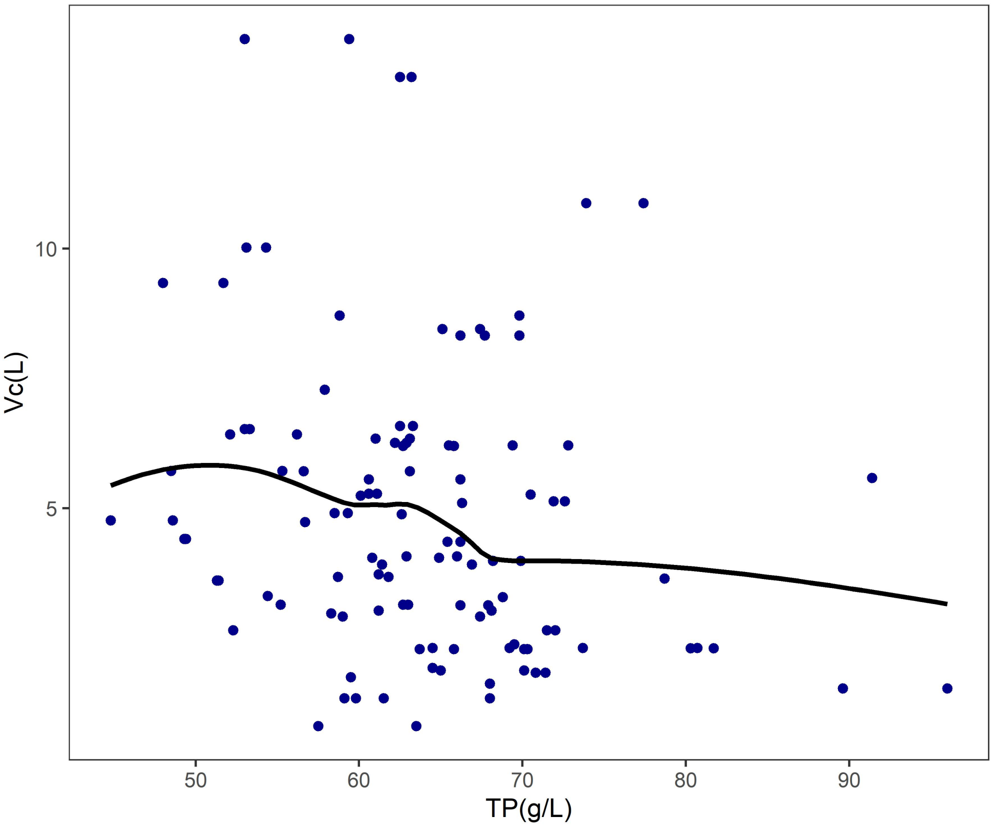

M1

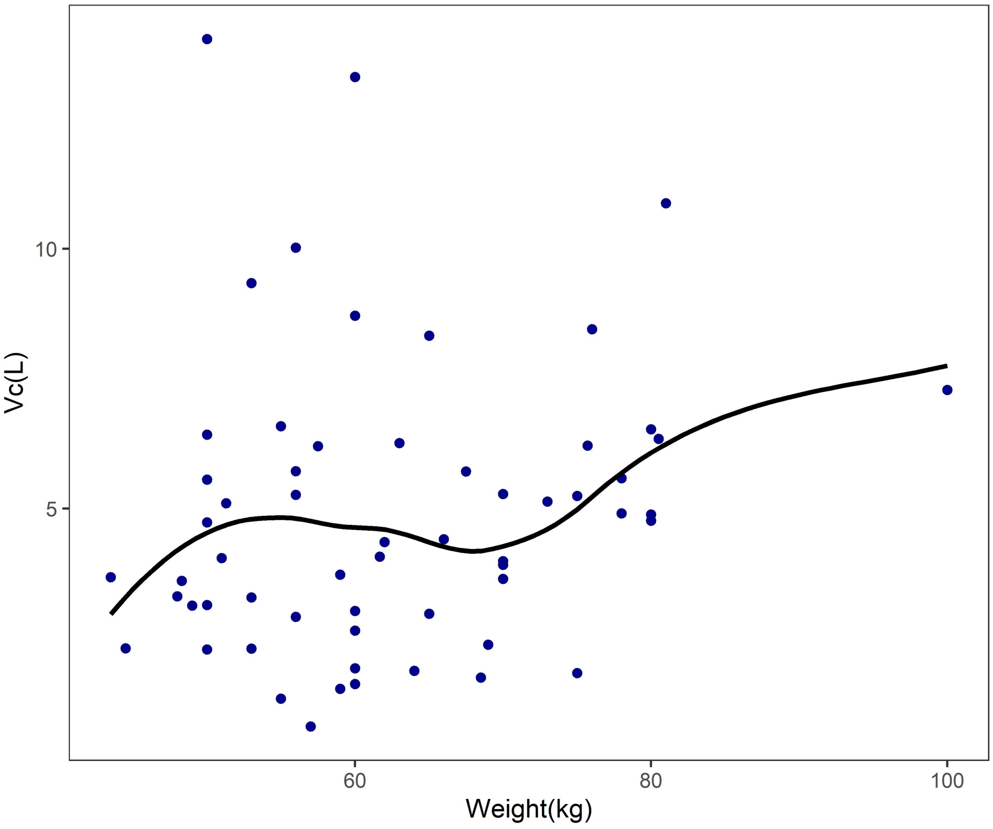

N1

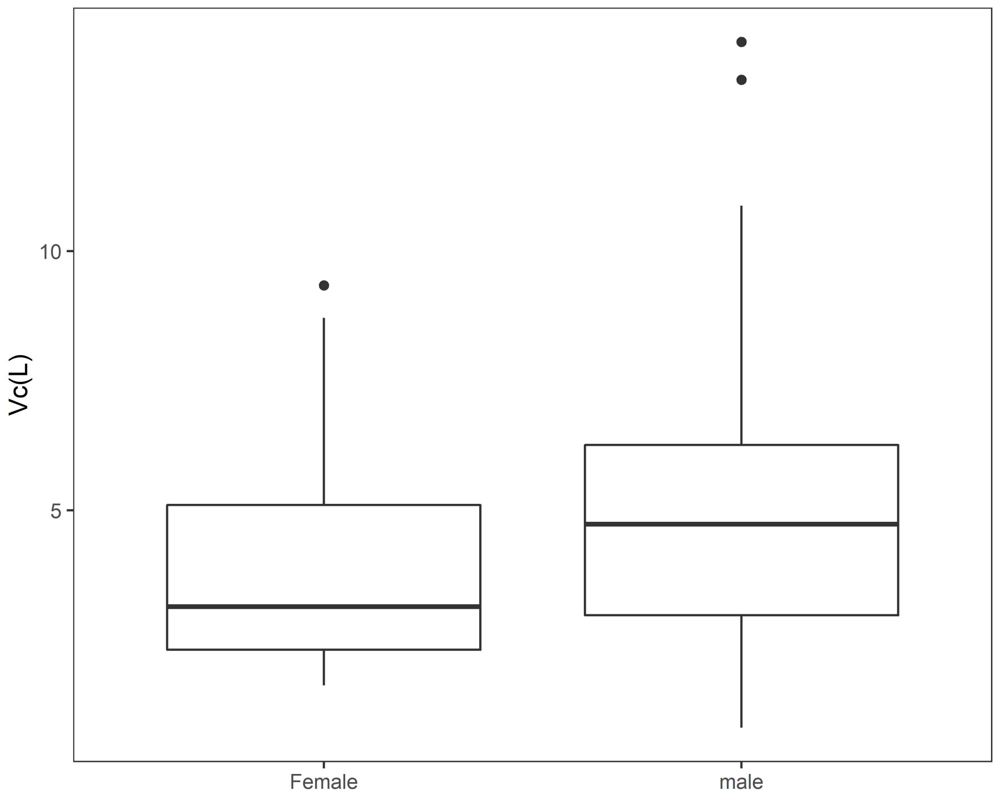

O1

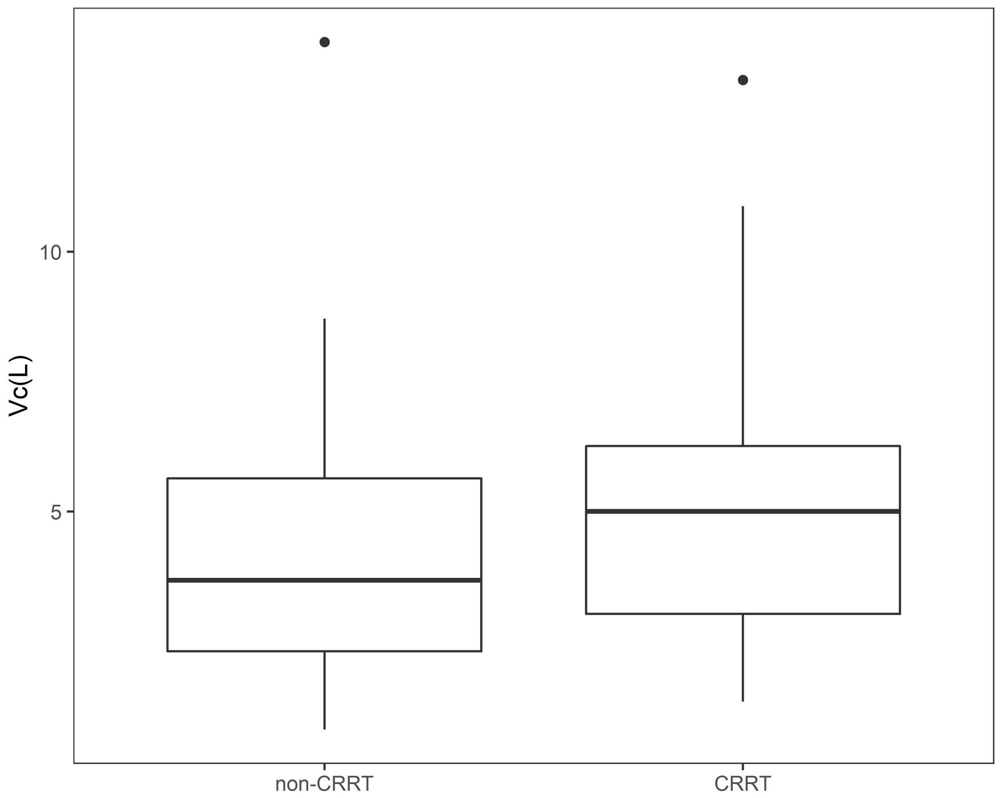

P1

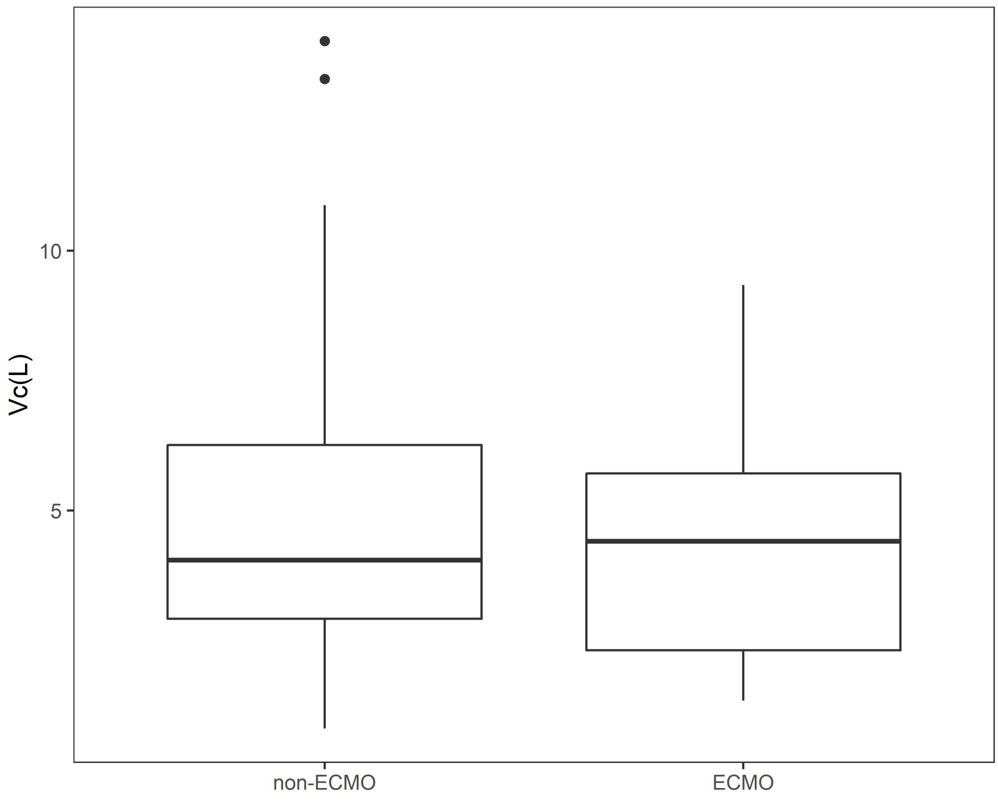

Q1

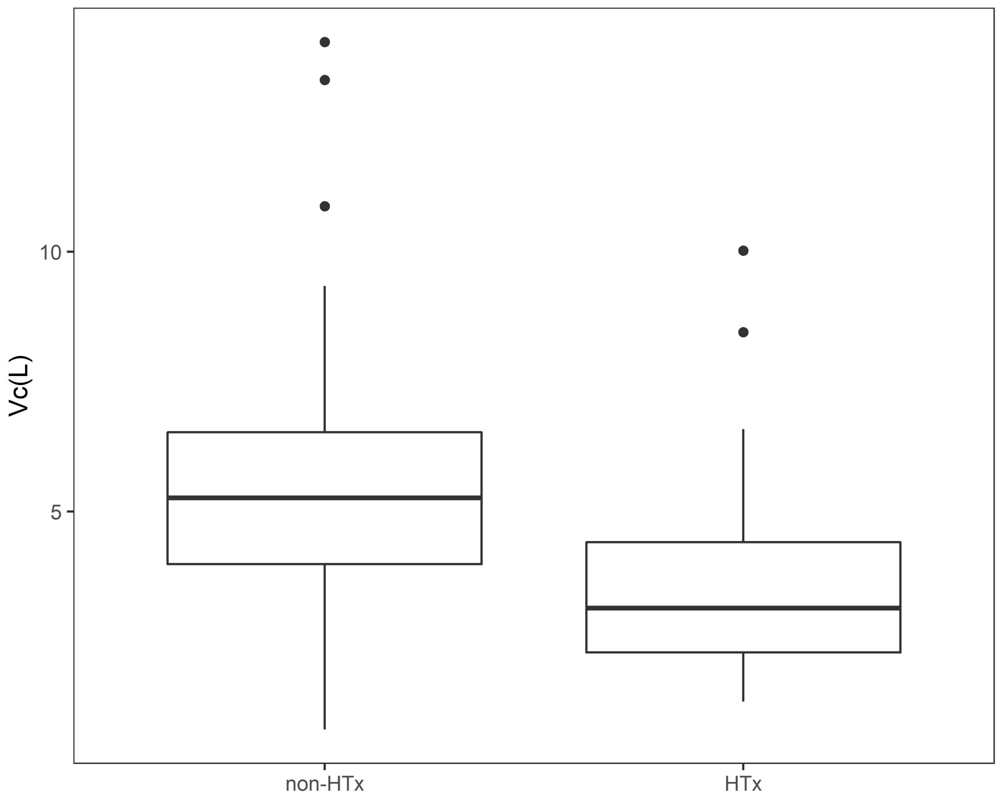

Figure S2 The relationship between the covariates and pharmacokinetics parameters (clearance (A-Q) and volume of the central compartment (A1-Q1))

CL, Clearance; Vc, volume of the central compartment; ALB Albumin; ALT, Alanine aminotransferase; AST, Aspartate aminotransferase; CLCR, Creatinine clearance; DBIL, Direct bilirubin; TBIL,Total bilirubin; PCT, Procalcitonin; PLT Platelet count; Scr Serum creatine; TP Total protein; CRRT Continuous renal replacement therapy; ECMO, extracorporeal membrane oxygenation ; HTx, heart transplantation.
